# Supplementary material for: Gender differences in the Italian academic landscape: Examining inequalities within the medical area in the last decade
Source: PLoS One. 2025 Jul 23;20(7):e0325705. doi: 10.1371/journal.pone.0325705 (PMC12286393; doi:10.1371/journal.pone.0325705)
Supplement: S1 File — (DOCX) [file pone.0325705.s001.docx]

**SUPPORTING INFORMATION**

**Table S1. Scientific-disciplinary sectors in the Medical Area for Italian University Research and Teaching**

| **Scientific-disciplinary sectors - SSDs** | | |
| --- | --- | --- |
| MED/01 | Statistica medica | Medical statistics |
| MED/02 | Storia della medicina | Medical history |
| MED/03 | Genetica medica | Medical genetics |
| MED/04 | Patologia generale | Experimental medicine and pathophysiology |
| MED/05 | Patologia clinica | Clinical pathology |
| MED/06 | Oncologia medica | Medical oncology |
| MED/07 | Microbiologia e microbiologia clinica | Microbiology and clinical microbiology |
| MED/08 | Anatomia patologica | Pathology |
| MED/09 | Medicina interna | Internal medicine |
| MED/10 | Malattie dell'apparato respiratorio | Respiratory diseases |
| MED/11 | Malattie dell'apparato cardiovascolare | Cardiovascular diseases |
| MED/12 | Gastroenterologia | Gastroenterology |
| MED/13 | Endocrinologia | Endocrinology |
| MED/14 | Nefrologia | Nephrology |
| MED/15 | Malattie del sangue | Blood diseases |
| MED/16 | Reumatologia | Rheumatology |
| MED/17 | Malattie infettive | Infectious diseases |
| MED/18 | Chirurgia generale | General surgery |
| MED/19 | Chirurgia plastica | Plastic surgery |
| MED/20 | Chirurgia pediatrica e infantile | Paediatric surgery |
| MED/21 | Chirurgia toracica | Thoracic surgery |
| MED/22 | Chirurgia vascolare | Vascular surgery |
| MED/23 | Chirurgia cardiaca | Heart surgery |
| MED/24 | Urologia | Urology |
| MED/25 | Psichiatria | Psychiatry |
| MED/26 | Neurologia | Neurology |
| MED/27 | Neurochirurgia | Neurosurgery |
| MED/28 | Malattie odontostomatologiche | Oral diseases and dentistry |
| MED/29 | Chirurgia maxillofacciale | Maxillofacial surgery |
| MED/30 | Malattie apparato visivo | Eye diseases |
| MED/31 | Otorinolaringoiatria | Otorhinolaryngology |
| MED/32 | Audiologia | Audiology |
| MED/33 | Malattie apparato locomotore | Musculoskeletal system diseases |
| MED/34 | Medicina fisica e riabilitativa | Physical and rehabilitation medicine |
| MED/35 | Malattie cutanee e veneree | Dermatological and venerological diseases |
| MED/36 | Diagnostica per immagini e radioterapia | Diagnostic imaging and radiotherapy |
| MED/37 | Neuroradiologia | Neuroradiology |
| MED/38 | Pediatria generale e specialistica | General and subspecialty paediatrics |
| MED/39 | Neuropsichiatria infantile | Child neuropsychiatry |
| MED/40 | Ginecologia e ostetricia | Obstetrics and gynaecology |
| MED/41 | Anestesiologia | Anaesthesiology |
| MED/42 | Igiene generale e applicata | Hygiene and public health |
| MED/43 | Medicina legale | Forensic medicine |
| MED/44 | Medicina del lavoro | Occupational medicine |
| MED/45 | Scienze infermieristiche generali, cliniche e pediatriche | Nursing sciences: general, clinical and paediatric |
| MED/46 | Scienze tecniche di medicina di laboratorio | Medical and biotechnology laboratory techniques |
| MED/47 | Scienze infermieristiche ostetrico-ginecologiche | Midwifery |
| MED/48 | Scienze infermieristiche e tecniche neuro-psichiatriche e riabilitative | Neuropsychiatric and rehabilitation nursing sciences |
| MED/49 | Scienze tecniche dietetiche applicate | Food sciences and dietetics |
| MED/50 | Scienze tecniche mediche applicate | Applied medical techniques |

**Table S2. Detailed 2023 data on academic positions, disaggregated by SSD**

| **SSD** | **Gender** | **Research Fellows** | **RTDA** | **RTDB** | **Tenured Researches** | **Associate Professors** | **Full Professors** | **Temporary Extraordinary Professor** |
| --- | --- | --- | --- | --- | --- | --- | --- | --- |
| MED/01 | Female | 22 | 90 | 70 | 118 | 268 | 114 | 7 |
|  | Male | 8 | 53 | 48 | 57 | 218 | 182 | 7 |
| MED/02 | Female | 4 | 14 | 10 | 60 | 67 | 21 |  |
|  | Male | 1 | 14 | 17 | 63 | 44 | 26 |  |
| MED/03 | Female | 47 | 100 | 61 | 266 | 356 | 110 | 1 |
|  | Male | 9 | 61 | 40 | 102 | 285 | 274 | 3 |
| MED/04 | Female | 117 | 399 | 178 | 1033 | 892 | 425 | 9 |
|  | Male | 37 | 156 | 121 | 400 | 726 | 766 | 22 |
| MED/05 | Female | 17 | 95 | 50 | 235 | 206 | 92 | 0 |
|  | Male | 3 | 26 | 12 | 116 | 117 | 172 | 3 |
| MED/06 | Female | 46 | 129 | 51 | 172 | 137 | 29 | 1 |
|  | Male | 14 | 104 | 62 | 192 | 352 | 305 | 20 |
| MED/07 | Female | 43 | 188 | 96 | 639 | 681 | 242 | 0 |
|  | Male | 19 | 77 | 59 | 290 | 399 | 340 | 10 |
| MED/08 | Female | 19 | 93 | 44 | 339 | 350 | 188 | 1 |
|  | Male | 11 | 87 | 48 | 298 | 566 | 516 | 8 |
| MED/09 | Female | 65 | 229 | 125 | 849 | 630 | 148 | 6 |
|  | Male | 30 | 209 | 175 | 1130 | 1883 | 1191 | 38 |
| MED/10 | Female | 17 | 45 | 18 | 98 | 118 | 43 | 0 |
|  | Male | 4 | 38 | 41 | 150 | 290 | 209 | 16 |
| MED/11 | Female | 29 | 90 | 46 | 254 | 218 | 42 | 0 |
|  | Male | 9 | 116 | 133 | 429 | 724 | 408 | 32 |
| MED/12 | Female | 23 | 79 | 34 | 109 | 169 | 41 | 1 |
|  | Male | 7 | 63 | 57 | 177 | 486 | 320 | 13 |
| MED/13 | Female | 61 | 161 | 76 | 212 | 325 | 81 | 0 |
|  | Male | 9 | 99 | 46 | 287 | 571 | 428 | 10 |
| MED/14 | Female | 15 | 47 | 25 | 89 | 66 | 23 | 6 |
|  | Male | 3 | 36 | 30 | 195 | 313 | 177 | 2 |
| MED/15 | Female | 75 | 150 | 39 | 267 | 216 | 33 | 7 |
|  | Male | 36 | 84 | 46 | 214 | 486 | 329 | 6 |
| MED/16 | Female | 20 | 101 | 41 | 148 | 156 | 29 | 0 |
|  | Male | 5 | 65 | 34 | 121 | 224 | 201 | 9 |
| MED/17 | Female | 28 | 53 | 28 | 210 | 185 | 57 | 0 |
|  | Male | 8 | 62 | 57 | 210 | 303 | 265 | 15 |
| MED/18 | Female | 16 | 92 | 52 | 525 | 257 | 57 | 5 |
|  | Male | 5 | 225 | 195 | 1776 | 2275 | 1151 | 33 |
| MED/19 | Female | 2 | 12 | 16 | 43 | 40 | 14 | 0 |
|  | Male | 1 | 29 | 48 | 148 | 230 | 211 | 5 |
| MED/20 | Female | 1 | 20 | 8 | 28 | 17 | 9 | 5 |
|  | Male | 2 | 16 | 24 | 79 | 167 | 133 | 6 |
| MED/21 | Female | 4 | 17 | 4 | 26 | 22 | 5 | 0 |
|  | Male | 0 | 23 | 23 | 106 | 326 | 168 | 5 |
| MED/22 | Female | 0 | 20 | 5 | 63 | 45 | 1 | 0 |
|  | Male | 2 | 54 | 35 | 180 | 309 | 201 | 3 |
| MED/23 | Female | 4 | 20 | 1 | 10 | 12 | 14 | 0 |
|  | Male | 2 | 26 | 28 | 241 | 313 | 242 | 12 |
| MED/24 | Female | 4 | 11 | 1 | 45 | 27 | 5 | 0 |
|  | Male | 6 | 85 | 54 | 379 | 452 | 342 | 5 |
| MED/25 | Female | 20 | 55 | 41 | 207 | 195 | 90 | 3 |
|  | Male | 13 | 98 | 75 | 254 | 360 | 297 | 14 |
| MED/26 | Female | 55 | 202 | 82 | 402 | 436 | 72 | 1 |
|  | Male | 31 | 222 | 116 | 509 | 840 | 614 | 6 |
| MED/27 | Female | 3 | 10 | 11 | 54 | 17 | 8 | 0 |
|  | Male | 5 | 41 | 42 | 228 | 393 | 224 | 11 |
| MED/28 | Female | 29 | 106 | 56 | 368 | 316 | 181 | 1 |
|  | Male | 19 | 210 | 169 | 724 | 1304 | 724 | 30 |
| MED/29 | Female | 3 | 14 | 7 | 42 | 29 | 4 | 0 |
|  | Male | 3 | 59 | 39 | 145 | 195 | 171 | 1 |
| MED/30 | Female | 14 | 78 | 25 | 287 | 98 | 16 | 0 |
|  | Male | 11 | 85 | 41 | 560 | 502 | 305 | 9 |
| MED/31 | Female | 10 | 27 | 22 | 67 | 64 | 4 | 0 |
|  | Male | 1 | 59 | 54 | 282 | 463 | 336 | 7 |
| MED/32 | Female | 3 | 17 | 7 | 98 | 71 | 13 | 0 |
|  | Male | 1 | 18 | 3 | 75 | 177 | 47 | 4 |
| MED/33 | Female | 4 | 13 | 11 | 42 | 51 | 6 | 0 |
|  | Male | 8 | 101 | 97 | 337 | 636 | 398 | 28 |
| MED/34 | Female | 7 | 31 | 18 | 74 | 100 | 51 | 0 |
|  | Male | 9 | 31 | 35 | 70 | 205 | 158 | 5 |
| MED/35 | Female | 21 | 77 | 57 | 195 | 203 | 84 | 5 |
|  | Male | 11 | 59 | 27 | 197 | 303 | 170 | 2 |
| MED/36 | Female | 23 | 95 | 45 | 357 | 301 | 81 | 1 |
|  | Male | 14 | 155 | 141 | 546 | 823 | 663 | 17 |
| MED/37 | Female | 0 | 13 | 9 | 25 | 46 | 10 | 0 |
|  | Male | 1 | 15 | 28 | 61 | 126 | 99 | 9 |
| MED/38 | Female | 55 | 173 | 87 | 534 | 495 | 97 | 3 |
|  | Male | 17 | 60 | 84 | 415 | 594 | 512 | 10 |
| MED/39 | Female | 15 | 66 | 49 | 163 | 152 | 42 | 1 |
|  | Male | 4 | 14 | 13 | 87 | 141 | 141 | 0 |
| MED/40 | Female | 16 | 69 | 42 | 484 | 293 | 64 | 4 |
|  | Male | 2 | 83 | 64 | 553 | 791 | 463 | 13 |
| MED/41 | Female | 8 | 31 | 26 | 315 | 148 | 55 | 6 |
|  | Male | 7 | 62 | 92 | 472 | 571 | 334 | 10 |
| MED/42 | Female | 51 | 155 | 89 | 530 | 551 | 283 | 5 |
|  | Male | 17 | 92 | 72 | 353 | 459 | 548 | 20 |
| MED/43 | Female | 13 | 43 | 39 | 277 | 245 | 86 | 6 |
|  | Male | 4 | 56 | 83 | 382 | 457 | 331 | 10 |
| MED/44 | Female | 13 | 43 | 34 | 171 | 148 | 34 | 0 |
|  | Male | 12 | 49 | 48 | 162 | 294 | 197 | 15 |
| MED/45 | Female | 16 | 28 | 19 | 37 | 126 | 43 |  |
|  | Male | 2 | 34 | 44 | 17 | 88 | 11 |  |
| MED/46 | Female | 42 | 182 | 84 | 235 | 415 | 96 | 1 |
|  | Male | 13 | 70 | 58 | 100 | 230 | 130 | 1 |
| MED/47 | Female |  | 9 |  | 22 | 13 | 1 |  |
|  | Male |  | 0 |  | 1 | 2 | 4 |  |
| MED/48 | Female | 5 | 27 | 15 | 37 | 57 | 28 | 0 |
|  | Male | 4 | 21 | 21 | 37 | 42 | 23 | 1 |
| MED/49 | Female | 27 | 94 | 51 | 93 | 153 | 51 | 0 |
|  | Male | 8 | 40 | 27 | 79 | 171 | 107 | 8 |
| MED/50 | Female | 25 | 134 | 60 | 122 | 144 | 63 | 0 |
|  | Male | 14 | 52 | 79 | 122 | 251 | 201 | 4 |

**Table S3. Detailed 2023 data on academic positions, disaggregated by region**

| **Region** | **Gender** | **Research Fellows** | **RTDA** | **RTDB** | **Tenured Researches** | **Associate Professors** | **Full Professors** | **Temporary Extraordinary Professor** |
| --- | --- | --- | --- | --- | --- | --- | --- | --- |
| Abruzzo | Female | 30 | 15 | 7 | 18 | 57 | 21 |  |
|  | Male | 8 | 13 | 21 | 15 | 65 | 64 |  |
| Basilicata | Female | 1 | 1 |  |  | 0 |  |  |
|  | Male | 0 | 0 |  |  | 1 |  |  |
| Calabria | Female | 11 | 25 | 14 | 2 | 32 | 11 |  |
|  | Male | 2 | 9 | 21 | 5 | 44 | 40 |  |
| Campania | Female | 88 | 102 | 51 | 24 | 114 | 58 |  |
|  | Male | 18 | 70 | 63 | 43 | 209 | 161 |  |
| Emilia-Romagna | Female | 197 | 56 | 52 | 40 | 145 | 39 | 2 |
|  | Male | 92 | 42 | 51 | 34 | 223 | 152 | 15 |
| Friuli Venezia Giulia | Female | 10 | 7 | 6 | 8 | 30 | 10 |  |
|  | Male | 10 | 3 | 9 | 18 | 64 | 27 |  |
| Lazio | Female | 125 | 127 | 66 | 156 | 259 | 72 | 0 |
|  | Male | 62 | 102 | 84 | 205 | 434 | 266 | 6 |
| Liguria | Female | 30 | 18 | 9 | 10 | 44 | 8 | 0 |
|  | Male | 11 | 19 | 21 | 9 | 64 | 46 | 2 |
| Lombardia | Female | 167 | 124 | 60 | 42 | 205 | 75 | 2 |
|  | Male | 69 | 105 | 110 | 52 | 370 | 313 | 15 |
| Marche | Female | 16 | 14 | 12 | 5 | 33 | 14 |  |
|  | Male | 1 | 12 | 18 | 9 | 37 | 37 |  |
| Molise | Female |  | 1 | 2 |  | 5 | 0 | 0 |
|  | Male |  | 7 | 4 |  | 13 | 10 | 4 |
| Piemonte | Female | 105 | 34 | 22 | 8 | 96 | 32 | 1 |
|  | Male | 42 | 23 | 38 | 14 | 126 | 78 | 0 |
| Puglia | Female | 23 | 32 | 9 | 26 | 46 | 21 | 0 |
|  | Male | 9 | 26 | 23 | 29 | 95 | 82 | 2 |
| Sardegna | Female | 10 | 18 | 8 | 17 | 37 | 8 |  |
|  | Male | 6 | 9 | 18 | 14 | 57 | 56 |  |
| Sicilia | Female | 17 | 40 | 33 | 56 | 95 | 48 |  |
|  | Male | 11 | 37 | 49 | 61 | 169 | 139 |  |
| Telematica | Female | 1 | 5 | 2 |  | 11 | 1 | 0 |
|  | Male | 0 | 0 | 1 |  | 6 | 6 | 5 |
| Toscana | Female | 185 | 68 | 35 | 41 | 110 | 36 | 0 |
|  | Male | 81 | 38 | 45 | 24 | 168 | 110 | 1 |
| Trentino Alto Adige | Female | 0 |  | 1 | 0 | 1 | 1 |  |
|  | Male | 1 |  | 3 | 1 | 7 | 6 |  |
| Umbria | Female | 26 | 5 | 5 | 16 | 26 | 5 | 0 |
|  | Male | 6 | 5 | 6 | 10 | 48 | 19 | 1 |
| Veneto | Female | 115 | 59 | 37 | 22 | 119 | 23 |  |
|  | Male | 33 | 45 | 40 | 14 | 171 | 110 |  |

**Figure S1. Temporal trends of gender disparities within Regions according to GCI**

**Figure S2. Temporal trends of gender disparities within Regions according to GDI**

**Figure S3. Temporal trends of gender disparities within SSD according to GCI**

**Figure S4. Temporal trends of gender disparities within SSD according to GDI**
