# Supplementary material for: Gender differences in the Italian academic landscape: Examining inequalities within the medical area in the last decade
Source: PLoS One. 2025 Jul 23;20(7):e0325705. doi: 10.1371/journal.pone.0325705 (PMC12286393; doi:10.1371/journal.pone.0325705)
Supplement: Attachment — Submitted filename: PONE-D-25-05019_R1_reviewer.pdf [file pone.0325705.s003.pdf]

PLOS ONE

Gender Differences in the Italian Academic Landscape: Examining Inequalities within the Medical Area in the Last Decade

--Manuscript Draft--

**Manuscript Number:**	PONE-D-25-05019R1
**Article Type:**	Research Article
**Full Title:**	Gender Differences in the Italian Academic Landscape: Examining Inequalities within the Medical Area in the Last Decade
**Short Title:**	Gender Differences in the Italian Academic Landscape
**Corresponding Author:**	Antonella Agodi<br>University of Catania: Universita degli Studi di Catania<br>Catania, ITALY
**Keywords:**	Gender, Italy, Academic research, Glass Ceiling Index, Health professionals
**Abstract:**	Gender equality remains a key focus in research and innovation policies. However, the academic sector still exhibits significant under-representation of women in leadership roles and horizontal segregation affecting career trajectories. This study examines gender inequalities in top positions and career progression within Italy's Medical Sciences sector (Area 06) from 2014 to 2023, through data from the Cineca portal, including details on academic roles, gender, region, and scientific-disciplinary sectors (SSDs). Gender disparity was evaluated using the Glass Ceiling Index (GCI) and the Glass Door Index (GDI). Data analysis up to 2023 shows a gender inversion in career progression: women dominate early career stages – accounted for 71.5% of research fellows and 57.1% of RTDA positions –, but men prevail in senior roles, with 61.8% associate professors and 71.8% full professors. This trend is consistent across most SSDs. Although temporal analysis indicates a reduction in gender disparities in top positions – with a GCI ranging from 2.3 in 2015 to 1.7 in 2023 - GCI values above one across all regions suggest persistent gender inequality. The GDI values, ranging from 1.3 in 2015 to 1.6 in 2023, exhibit considerable variability; however, overall, the disparities have worsened by 2023. These findings underscore the need for concrete measures to enhance equity and inclusivity in academia. Developing and implementing initiatives, programs, and policies that guarantee equal opportunities and resources for all researchers, irrespective of gender, is essential.
**Order of Authors:**	Roberta Magnano San Lio
	Mara Morini
	Enrico Di Rosa
	Alessandra Sinipoli
	Virginia Casigliani
	Manuela Martella
	Andrea Maugeri
	Martina Barchitta
	Maria Carmela Agodi
	Ilenia Picardi
	Antonella Agodi
**Response to Reviewers:**	Dear Editor,<br>We would like to thank you and the reviewers for the time and effort dedicated to evaluating our manuscript titled " Gender Differences in the Italian Academic Landscape: Examining Inequalities within the Medical Area in the Last Decade". We greatly appreciate the insightful and constructive comments, which have helped us improve the clarity, structure, and scientific value of our work.<br>Below, we provide a point-by-point response to each reviewer’s comments. All

changes have been incorporated in the revised version of the manuscript, which we resubmit for your consideration.

Reviewer #1
Comment (C): Although the study is well conducted, and the manuscript written in an intelligible way, there are parts that would make it better for the reader to understand methods and results better. I have added those explicitly in the attached file. Thanks.

Answer (A): We are grateful to Reviewer 1 for this comment and for the suggested improvements. We have revised the manuscript accordingly. Some parts of the introduction have been removed in line with feedback from other reviewers. The "Integrazione del decreto del Ministro dell'istruzione, dell'università e della ricerca 1 settembre 2016, n. 662" could not be translated into English, as it refers to a specific Italian legal document. In the Results section, we chose not to include percentages for all specialties with male predominance, to maintain clarity and readability. However, we have made the table more reader-friendly, as suggested. In the Discussion, we added a comment and reference on the challenges women face in balancing family and academic careers, particularly regarding decisions around motherhood. We also removed redundant sentences to avoid overlap with the Introduction. Lastly, references were not included in the Conclusions section, in line with standard academic conventions.

Reviewer #2
C: This is potentially an interesting manuscript in that there is evidence that fewer women are making it into the higher academic positions related to medicine and all of its related subspecialties across Italy. There is also some evidence that there is also some regional variation in these gender ratios as well.
A: We thank Reviewer 2 for this overall positive assessment.
C: There is an absence of numerical data elements in the results section of the abstract.
A: We apologize for the lack of clarity in the abstract. Numerical elements have now been included in the revised abstract to enhance its informative value.
C: An extremely long Introduction section which needs to be condensed down with some of the relevant information being used for the Discussion section (to prevent repeating the same information in the Discussion section, which is apparent in places).
A: We fully agree and have significantly revised and shortened the Introduction. Relevant content has been redistributed or removed to reduce repetition with the Discussion section.
C: There is a failure to provide the summary data for the actual numbers of females versus males for the various medical subspecialties with instead only the data for the ratios being provided. It would be useful to provide this summary data along with the gender ratios so that the reader could more readily ascertain as to where real progress is being made (ie within either the subspecialties or the regions with higher numbers of medical professionals overall). This would be pertinent to Figure 1, Figure 2 and Figure 5. As the authors allude to on lines 288-289 low numbers of medical professionals in some regions mean that the results of the GDI values have to be treated with caution. Plus, there is also the issue with missing data elements for the GDI and the GCI (see below).
A: We apologize if this part of our analysis was unclear. As requested, we have now included absolute numbers for each academic position for the year 2023 at the beginning of the Results section. Additionally, in the Supplementary Information, we have provided two tables stratified by SSD and by region. Data for other years remain available through the Cineca portal (https://cercauniversita.mur.gov.it).
However, we believe that including absolute numbers for the time trend graphs would reduce readability without adding substantial value. The use of proportions, ratios, and calculated indices provides a clearer and more standardized interpretation of gender disparities, which might otherwise be obscured by raw numbers.
C: A number of the Tables/Figures are too crowded/contain too much data to appear in the main manuscript. For eg Table 1, Figures 7-10.
A: We appreciate this suggestion. To improve the manuscript's readability, we have moved Table 1 and Figures 7–10 to the Supplementary Information.
C: Plus of note there seem to be missing data elements for both the GCI and the GDI in Figures 7-10. Are these missing data elements excluded from any further consideration in the results? Plus does this not limit the validity of the GCI/GDI

calculations? Hence this raises the question as to how reliable the summary data that is contained in the graphics in Figure 3 and Figure 4 actually is. It seems that the only data which can be reliably depicted/summarized for either the CGI or GDI is where there is a complete set for either the GCI or GDI per year either for the subspecialty or for the region of interest. It is this subset of the data that needs to be focused on in the results (with a median and range being reported of either the overall GCI or GDI score per year for all of the study group and then for each region-if there is a complete set of data)

A: Thank you for highlighting this important point. We would like to clarify that the elements in question are not technically "missing." Rather, in some instances, the data available did not meet the conditions necessary for calculating the indices. As clarified in the revised Methods and Results sections, the GCI and GDI could not be computed in regions or SSDs where key academic roles were absent (e.g., no full professors or no RTDA/RTDB positions).

We now specify in the manuscript that summary statistics (e.g., medians and ranges) are presented only for subsets with complete data. These limitations have also been explicitly acknowledged in the revised Discussion section.

Reviewer #3

C: The aim of this manuscript is to examine gender inequalities in top positions and career progression within Italy's Medical Sciences sector (Area 06) from 2014 to 2023, through data from the Cineca portal, including details on academic roles, gender, region, and scientific-disciplinary (SSDs). This manuscript is well-structured, informative, and supported by relevant data and sources. However, there are a few suggestions for improvement regarding clarity, and conciseness. For these reasons, the manuscript requires major changes. Please find below an enumerated list of comments on my review of the manuscript.

A: We thank Reviewer 3 for this kind and constructive evaluation.

C: The authors should provide a list of abbreviations.

A: We have now included a list of abbreviations at the end of the manuscript, before the References section.

C: The term “gender” indicates the identities of male, female, and gender-diverse populations in a social context. The World Health Organization’s (WHO) definition of gender refers to “the roles, behaviors, activities, attributes, and opportunities that any society considers appropriate for girls and boys, and women and men”(see, for reference: https://doi.org/10.3390/healthcare11060828). This is the major concern of this manuscript: this manuscript will benefit from providing an organic definition of the term “gender”, in according to WHO and recent scientific evidence on this topic.

A: We thank the reviewer for raising this point. However, we are unable to adopt the broader WHO definition of “gender” as our data source (the Cineca portal) categorizes individuals strictly by legal sex: male (M) and female (F). As such, our use of the term “gender” reflects this binary classification and does not include self-identified gender identities.

C:, there is a strong connection between gender differences and STEM (Science, Technology, Engineering, and Mathematics), which manifests in multiple ways, including representation, career advancement, biases, and societal perceptions (see, for reference: https://doi.org/10.1080/2331186X.2024.2439655).

A: We appreciate this suggestion and have revised the Introduction accordingly to include this important aspect, along with the suggested reference.

C: As regards the originality and strengths of this manuscript, this is a significant contribute to the ongoing research on this topic, as it provides a broad overview of gender disparities in academia, specifically within the European and Italian contexts. Overall, the contents are rich, and the authors also give their deep insight for some works. Moreover, the discussion of this manuscript effectively connects gender disparities to structural and cultural barriers.

A: We sincerely thank Reviewer 3 for the encouraging feedback and support for our study.

C: However, major concerns of this manuscript are with the introductive section: for these reasons, I have major comments for this section, for improvement before acceptance for publication. The article is accurate and provides relevant information on the topic and I have some major points to make, that may help to improve the quality of the current manuscript and maximize its scientific impact. I would accept this manuscript if the comments are addressed properly.

A: We have thoroughly revised the Introduction based on all reviewers’ feedback, improving its clarity, focus, and conciseness.

Powered by Editorial Manager® and ProduXion Manager® from Aries Systems Corporation

	Reviewer #4<br>C: The manuscript is scientifically sound. There is no mention of getting any IRB approval. It seems like its not needed or applicable here. Kindly check with the authors regarding getting any IRB approval.<br>A: We confirm that ethical approval was not required for this study, as all data were obtained from the publicly accessible Cineca portal. The data are anonymized and presented in aggregated form, in compliance with ethical research standards.<br>Once again, we sincerely thank all reviewers for their valuable contributions. We hope the revised version meets the journal's standards and look forward to your feedback.<br>Kind regards,<br>Antonella Agodi
**Additional Information:**	
**Question**	**Response**
**Financial Disclosure**<br><br>Enter a financial disclosure statement that describes the sources of funding for the work included in this submission. Review the submission guidelines for detailed requirements. View published research articles from *PLOS ONE* for specific examples.<br><br>This statement is required for submission and **will appear in the published article** if the submission is accepted. Please make sure it is accurate.<br><br>**Funded studies**<br>Enter a statement with the following details:<br>• Initials of the authors who received each award<br>• Grant numbers awarded to each author<br>• The full name of each funder<br>• URL of each funder website<br>• Did the sponsors or funders play any role in the study design, data collection and analysis, decision to publish, or preparation of the manuscript?<br><br>Did you receive funding for this work?	The author(s) received no specific funding for this work.
**Competing Interests**<br><br>Use the instructions below to enter a competing interest statement for this submission. On behalf of all authors, disclose any competing interests that could be perceived to bias this work—acknowledging all financial support and any other relevant financial or non-	The authors have declared that no competing interests exist

financial competing interests.<br><br>This statement is required for submission and **will appear in the published article** if the submission is accepted. Please make sure it is accurate and that any funding sources listed in your Funding Information later in the submission form are also declared in your Financial Disclosure statement.<br><br>View published research articles from *PLOS ONE* for specific examples.<br><br>**NO authors have competing interests**<br><br>Enter: *The authors have declared that no competing interests exist.*<br><br>**Authors with competing interests**<br><br>Enter competing interest details beginning with this statement:<br><br>*I have read the journal's policy and the authors of this manuscript have the following competing interests: [insert competing interests here]*<br><br>* typeset	
**Ethics Statement**<br><br>Enter an ethics statement for this submission. This statement is required if the study involved:<br><br>• Human participants<br>• Human specimens or tissue<br>• Vertebrate animals or cephalopods<br>• Vertebrate embryos or tissues<br>• Field research<br><br>Write "N/A" if the submission does not require an ethics statement.<br><br>General guidance is provided below.<br>Consult the submission guidelines for	N/A

detailed instructions. **Make sure that all information entered here is included in the Methods section of the manuscript.**<br><br>**Format for specific study types**<br><br>**Human Subject Research (involving human participants and/or tissue)**<br>• Give the name of the institutional review board or ethics committee that approved the study<br>• Include the approval number and/or a statement indicating approval of this research<br>• Indicate the form of consent obtained (written/oral) or the reason that consent was not obtained (e.g. the data were analyzed anonymously)<br><br>**Animal Research (involving vertebrate animals, embryos or tissues)**<br>• Provide the name of the Institutional Animal Care and Use Committee (IACUC) or other relevant ethics board that reviewed the study protocol, and indicate whether they approved this research or granted a formal waiver of ethical approval<br>• Include an approval number if one was obtained<br>• If the study involved *non-human primates*, add *additional details* about animal welfare and steps taken to ameliorate suffering<br>• If anesthesia, euthanasia, or any kind of animal sacrifice is part of the study, include briefly which substances and/or methods were applied<br><br>**Field Research**<br><br>Include the following details if this study involves the collection of plant, animal, or other materials from a natural setting:<br>• Field permit number<br>• Name of the institution or relevant body that granted permission	
**Data Availability**<br><br>Authors are required to make all data underlying the findings described fully available, without restriction, and from the time of publication. PLOS allows rare	Yes - all data are fully available without restriction

exceptions to address legal and ethical concerns. See the PLOS Data Policy and FAQ for detailed information.<br><br>A Data Availability Statement describing where the data can be found is required at submission. Your answers to this question constitute the Data Availability Statement and **will be published in the article**, if accepted.<br><br>**Important:** Stating 'data available on request from the author' is not sufficient. If your data are only available upon request, select 'No' for the first question and explain your exceptional situation in the text box.<br><br>Do the authors confirm that all data underlying the findings described in their manuscript are fully available without restriction?	
**Describe where the data may be found in full sentences. If you are copying our sample text, replace any instances of XXX with the appropriate details.**<br><br>• If the data are **held or will be held in a public repository**, include URLs, accession numbers or DOIs. If this information will only be available after acceptance, indicate this by ticking the box below. For example: *All XXX files are available from the XXX database (accession number(s) XXX, XXX.*).<br>• If the data are all contained **within the manuscript and/or Supporting Information files**, enter the following: *All relevant data are within the manuscript and its Supporting Information files.*<br>• If neither of these applies but you are able to provide **details of access elsewhere**, with or without limitations, please do so. For example:<br><br>*Data cannot be shared publicly because of [XXX]. Data are available from the XXX Institutional Data Access / Ethics Committee (contact via XXX) for researchers who meet the criteria for*	The data underlying the results presented in the study are available from the corresponding author

access to confidential data.<br><br>*The data underlying the results presented in the study are available from (include the name of the third party and contact information or URL).*<br>• This text is appropriate if the data are owned by a third party and authors do not have permission to share the data.<br><br>* typeset	
Additional data availability information:	

Powered by Editorial Manager® and ProduXion Manager® from Aries Systems Corporation

Manuscript Click here to access/download;Manuscript;Manuscript.docx

Gender Differences in the Italian Academic Landscape: Examining Inequalities within the Medical Area in the Last Decade

Magnano San Lio Roberta [1], Morini Mara [2], Di Rosa Enrico [2,3], Sinopoli Alessandra [2,3], Casigliani Virginia [2,4], Martella Manuela [2,5], Maugeri Andrea [1], Barchitta Martina [1], Agodi Maria Carmela [6], Picardi Ilenia [6], Agodi Antonella [1, 2*]

[1] Department of Medical and Surgical Sciences and Advanced Technologies "GF Ingrassia", University of Catania, 95123 Catania, Italy

[2] Italian Society of Hygiene, Preventive Medicine, and Public Health – SItI - Gender Prevention Working Group

[3] Department of Prevention, Local Health Authority Roma 1, 00193 Rome, Italy

[4] Department of Translational Research and of New Surgical and Medical Technologies, University of Pisa, Pisa, Italy

[5] Department of Public Health Science and Pediatrics, University of Turin, 10126 Torino, Italy

[6] Department of Political Science, University of Naples Federico II, Naples, Italy

on behalf of the Gender Prevention Working Group, SItI

Author’s note

*Corresponding author: Antonella Agodi antonella.agodi@unict.it

Abstract

Gender equality remains a key focus in research and innovation policies. However, the academic sector still exhibits significant under-representation of women in leadership roles and horizontal segregation affecting career trajectories. This study examines gender inequalities in top positions and career progression within Italy's Medical Sciences sector (Area 06) from 2014 to 2023, through data from the Cineca portal, including details on academic roles, gender, region, and scientific-disciplinary sectors (SSDs). Gender disparity was evaluated using the Glass Ceiling Index (GCI) and the Glass Door Index (GDI). Data analysis up to 2023 shows a gender inversion in career progression: women dominate early career stages, but men prevail in senior roles. This trend is consistent across most SSDs. Although temporal analysis indicates a reduction in gender disparities in top positions, GCI values above one across all regions suggest persistent gender inequality. The GDI values varied, but generally disparities worsened by 2023. These findings underscore the need for concrete measures to enhance equity and inclusivity in academia. Developing and implementing initiatives, programs, and policies that guarantee equal opportunities and resources for all researchers, irrespective of gender, is essential.

1. Introduction

Gender inequality remains a persistent issue in various professional fields, and academia is no exception [1, 2]. Despite regulatory interventions and gender mainstreaming policies, gender segregation ~~remains~~ persists, leading to the continued underrepresentation of women in top positions [3]. The European context provides a broader framework for analysing these issues, as gender disparity in academia is a well-documented phenomenon across the continent. Despite various initiatives and policies aimed at promoting gender equality, many European countries still exhibit substantial gender gaps in higher education and research. According to the European Commission's "She Figures 2021" report, women remain underrepresented in scientific and technical fields, and their progression to senior academic roles is significantly hindered compared to their male counterparts [4]. Specifically, women face significant barriers in achieving gender parity in research and innovation. Although women now represent 48% of doctoral graduates in Europe, their representation decreases significantly in advanced research roles and leadership positions. Moreover, disparities are particularly evident in STEM (science, technology, engineering, and mathematics) fields, where women remain a minority in both education and employment. For instance, in 2018, women accounted for only 32% of researchers in natural sciences and engineering. Financial disparities also persist, as women researchers are less likely to receive competitive grants or lead major projects. According to the *She Figures 2021* report, women comprised only 24% of applicants for European Research Council grants between 2014 and 2020, with lower success rates compared to men in several countries. These gaps reflect structural and cultural barriers, including unconscious biases, work-life balance challenges, and limited access to mentorship and networking opportunities. Addressing these disparities requires comprehensive approaches, such as targeted funding for women-led research, policies supporting work-life balance, and initiatives fostering inclusivity in traditionally male-dominated disciplines. In Italy, these disparities are evident in numerous aspects of academic life, including hiring practices, career progression, and access to research funding [5].

Recent research and reports indicate that, while women constitute a significant proportion of university graduates in Italy, their presence still dwindles at higher academic ranks [4, 6]. This trend mirrors the broader European pattern where, despite increasing numbers of female PhD graduates, the proportion of women in full professorship positions remains disproportionately low. Thus, gender inequalities manifest in the challenges women face in attaining top management and leadership positions in scientific institutions. These inequalities also include gender-based differentiation throughout academic career paths in the current Italian university system, beginning from the recruitment stages [7-10]. Recent studies have revealed that, despite women often having more distinguished graduate and post-graduate careers and earning degrees faster than men, especially in STEM fields, they are predominantly found in temporary positions in academia[11]. The percentage of female researchers hired on a permanent basis is significantly lower compared to their male counterparts, as it also happens at the European level. The Gender Equality Strategy 2020-2025 by the European Commission highlights the need for continued action to bridge the gender gaps that persist across multiple sectors, particularly in academia and research. One of its key objectives is to promote gender balance in decision-making processes, ensuring that women have equal representation in leadership roles within academic and scientific institutions. This includes initiatives to increase female participation in research leadership and decision-making bodies, as well as supporting women's access to funding, mentorship, and networks that have traditionally been dominated by men. Additionally, the strategy emphasizes the importance of ensuring equal economic independence for both women and men. This includes measures to tackle the gender pay gap, improve access to funding for women researchers, and provide support for women to advance in their careers without being hindered by unequal access to opportunities. The strategy also stresses the need for policies that help combat unconscious biases and structural barriers that hinder women's academic progression. These barriers include work-life balance challenges, the lack of flexible working hours, and ingrained cultural norms that perpetuate gender stereotypes. The strategy also calls for gender-responsive research and policies, which would involve integrating gender perspectives into all stages

of research and policymaking. This would ensure that gender equality is not only a theoretical goal but is embedded into practice, helping to address the systemic inequalities that limit women's success in scientific and academic careers. In practice, these measures have already been incorporated into the Horizon Europe program, which dedicates specific calls for proposals aimed at promoting gender equality in research teams. Furthermore, efforts are being made to enhance the visibility of female role models in academia, which can provide inspiration and guidance to younger generations of women scientists. Ultimately, the Gender Equality Strategy 2020-2025 aims to create an ecosystem where women and men have equal opportunities to thrive in all aspects of academic and scientific life, contributing to a more inclusive and innovative Europe [12, 13]. In Italy, academic careers in research and teaching are structured into several roles: Full Professor, Associate Professor, Researcher (a position being phased out), Fixed-term Researcher Type B (RTDB) as per Art. 24, para. 3, letter b of Law 240/2010, Fixed-term Researcher Type A (RTDA) as per Art. 24, para. 3, letter a of Law 240/2010, and Research Fellow.

RTDB positions involve a non-renewable three-year contract, but researchers can transition directly to Associate Professor if they possess the National Scientific Qualification and receive a positive evaluation from the university (it is a tenure track position). RTDA positions have contract lasting three years, renewable for an additional two years. Research fellows have contracts ranging from a minimum of one year to a maximum of three years, with a cumulative limit of six years for an individual in this role. As per the MIUR decree "Integrazione del decreto del Ministro dell'istruzione, dell'università e della ricerca 1 settembre 2016, n. 662, recante la tabella di corrispondenza tra posizioni accademiche italiane ed estere ai sensi dell'articolo 18, comma 1, lettera b), della Legge 30 dicembre 2010, n. 240.", the alignment of Italian and foreign academic positions stipulates that full professors correspond to GRADE A, associate professors to GRADE B, RTDB to GRADE C, and RTDA to GRADE D [14], as these GRADES are defined in the SHE Figures Report classification [4].

In addition to the roles mentioned, the position of Temporary Extraordinary Professor (as per Art. 1, para. 12 of Law 230/2005) remains in effect. This role involves a three-year contract that can be renewed for an additional three years and is funded by external entities for research activities. It is reserved for those who have obtained eligibility for the full professorship position or for individuals with high scientific and professional qualifications. Holders of these positions are entitled, for the duration of the contract, to the legal and economic treatment of full professors, with possible additional economic supplements, if provided for by the agreement [15].

The academic landscape in Italy has faced growing scrutiny over gender disparities in recent years, particularly within the medical field (Area 06 – Medicine). This study seeks to comprehensively analyse gender inequalities in academic medicine over the past decade, focusing on the period following the Gelmini Reform (Law 240/2010). This reform fundamentally reshaped the Italian academic system by introducing two fixed-term positions—the fixed-term contract A (RTDA) and the tenure-track fixed-term contract B (RTDB)—while abolishing the previously permanent role of researcher.

The primary aim of this study is to examine how these structural changes have influenced gender representation across different academic roles within medicine. It also aims to identify disparities across scientific-disciplinary sectors (Settori Scientifico-Disciplinari, SSD) and geographic regions in Italy, shedding light on regional and sector-specific dynamics that impact gender equity in academic career progression. By doing so, the study provides a nuanced understanding of the systemic challenges and opportunities for promoting gender parity in Italian academic medicine.

2. Materials and methods

The study analysed comprehensive data on academic roles spanning the years 2014 to 2023, sourced from the Cineca portal (https://cercauniversita.mur.gov.it/php5/docenti/cerca.php?SESSION). This dataset included detailed records on academic positions, encompassing research fellows, fixed-term

researchers (RTDA, RTDB), tenured researchers, associate professors, full professors, and temporary extraordinary professors. Each record provided information on academic roles, gender, affiliated university, region, and scientific-disciplinary sectors (SSD). Notably, data for research fellows were limited to the year 2023, while data for other positions spanned the entire period under study.

Certain categories of researchers were excluded from this analysis to ensure consistency and clarity. Specifically, data on full-time fixed-term researchers introduced under Law 79/2022, which replaced research fellowships in 2022, as well as researchers employed under Art. 1, para. 14, Law 230/05 prior to the differentiation into RTDA and RTDB categories, and data on non-tenured researchers, were omitted. This exclusion was necessary to maintain a uniform approach to analysing academic roles and to focus on positions with clear and consistent categorizations over the period.

For each academic role, data were initially examined by calculating gender proportions and the female-to-male ratio, providing insights into the distribution of men and women across various positions. To assess gender disparities, particularly in senior academic positions and recruitment pathways, the Glass Ceiling Index (GCI) was utilized. The GCI serves as a relative measure, comparing the proportion of women in academia at all levels (grades A, B, C, and D) to their representation in top academic positions (grade A) for a given year.

The GCI is computed using key gender-disaggregated variables for a given year Y, as follows:

- F_{AY} : Number of women full professors.
- M_{AY}: Number of men full professors.
- F_{BY}: Number of women associate professors.
- M_{BY}: Number of men associate professors.
- F_{CY} and F_{DY}: Number of women researchers (RTDB and RTDA, respectively).
- M_{CY} and M_{DY}: Number of men researchers (RTDB and RTDA, respectively).

These variables allow for a detailed examination of gender distribution across the academic hierarchy, from researchers to senior professors. The GCI quantitatively evaluates the extent to which women are underrepresented in higher academic positions relative to their overall presence in academia.

According to the She Figures Report, the GCI is defined as follows [16]:

$$GCI = (F_{AY} + F_{BY} + F_{CY} + F_{DY}) / (F_{AY} + F_{BY} + F_{CY} + F_{DY} + M_{AY} + M_{BY} + M_{CY} + M_{DY}) \,/\, F_{AY} / (F_{AY} + M_{AY})$$

Where the numerator calculates the proportion of women among the total academic workforce (all grades combined: A, B, C, and D), accounting for both female (F_{AY}, F_{BY}, $F_{CY,}$ and F_{DY}) and male (M_{AY}, M_{BY}, $M_{CY,}$ and M_{DY}) staff. The denominator calculates the proportion of women at the grade A level (F_{AY}) relative to the total number of grade A professors (F_{AY} and M_{AY}). Accordingly, the GCI is a continuous metric ranging from 0 to infinity, providing insights into gender disparities in achieving the highest academic ranks. A GCI of 1 indicates gender parity in promotion opportunities. Women and men have equal chances of being promoted to grade A (Full Professor), reflecting a balanced academic system. A GCI below 1 suggests that women are overrepresented at grade A relative to their overall presence in academia. This scenario is rare and might occur in specific disciplines or contexts where targeted policies or historical trends have favoured women's advancement. A GCI above 1 reveals a glass ceiling effect, where women are underrepresented in grade A positions compared to their overall representation in academia. The higher the GCI value, the more significant the barrier for women in advancing to Full Professor roles.

The more innovative part of the analysis is the one quantitatively analysing gender disparities in the recruitment phase, where the effects of the introduction of the fixed term positions RTDA and RTDB deploys its effects. To this aim, we employed th[illegible]DI, an index recently introduced to reveal and measure the gender bias in academic recruitment [17]. It is calculated as the ratio between two percentages: the percentage of women in temporary research positions ($PW_{\leq D}$) and the percentage of women in positions that lead to stable academic careers (PW_{D}). This index provides insight into the progression of women from temporary roles to more permanent, stable academic positions. Specifically:

$$GDI = PW_{\leq D,Y} / PW_{DY} = F_{\leq D,Y} / F_{\leq D,Y} + M_{\leq D,Y} \,/\, F_{DY} / F_{DY} + M_{DY}$$

where F_{DY} (M_{DY}) denotes the number of women (men) in the position leading to stable academic roles in year Y, and $F_{\leq D,Y}$ ($M_{\leq D,Y}$) represents the number of women (men) in temporary research positions and the position leading to academic roles in year Y. Specifically, we computed the GDI value using the RTDB position as the initial stable academic role. Briefly, based on the relative presence of women in temporary research roles and positions leading to academic roles, the GDI measures the fraction that has attained stabilization. Like the GCI, the GDI ranges from 0 to infinity. A GDI equal to or less than 1 indicates that the percentage of women in the tenure track position (RTDB) is greater or equal compared to their percentage in temporary positions (RTDA). Conversely, a GDI above 1 signals the presence of a glass door that restricts women's entry, reflecting a recruitment process towards permanent positions in academia biased against women. The higher the GDI value, the stronger the impact of this glass door on entry into academia.

In 2023, our analysis employed a variety of methodological approaches to comprehensively investigate gender representation across academic levels. Descriptive statistics were used to calculate the percentages of males and females at each academic rank, alongside the computation of the GDI and the Glass Ceiling Index GCI to quantitatively measure disparities in recruitment and progression. To visualize these disparities, we utilized scissor diagrams to depict gender distributions across academic levels, illustrating the divergence in career trajectories between men and women. Geographic variations in gender disparities were analysed through choropleth maps, which displayed the GDI and GCI indices across Italy, providing a spatial representation of regional differences (i.e., the region Valle d'Aosta is not present because it does not host any university). The temporal dimension of gender disparities was explored using a longitudinal analysis covering the period from 2014 to 2023. This analysis employed line graphs to examine changes in GDI, GCI, and the female-to-male ratio over time, allowing for the identification of trends and shifts across the decade. Heatmaps were further applied to capture the evolution of GDI and GCI across scientific-disciplinary sectors (SSDs) and Italian regions, enabling a granular understanding of gender disparities within specific fields and locations. The SSD classification used in this analysis was aligned with the official

categorizations provided by the Italian National University Council (CUN; https://www.cun.it/uploads/storico/settori_scientifico_disciplinari_english.pdf), as detailed in **Table 1**. Together, these methodological tools provided a robust framework for analysing and visualizing the complex dynamics of gender representation and disparities in academia.

Table 1. Scientific-disciplinary sectors in the Medical Area for Italian University Research and Teaching

**Scientific-disciplinary sectors - SSDs**		
MED/01	Statistica medica	Medical statistics
MED/02	Storia della medicina	Medical history
MED/03	Genetica medica	Medical genetics
MED/04	Patologia generale	Experimental medicine and pathophysiology
MED/05	Patologia clinica	Clinical pathology
MED/06	Oncologia medica	Medical oncology
MED/07	Microbiologia e microbiologia clinica	Microbiology and clinical microbiology
MED/08	Anatomia patologica	Pathology
MED/09	Medicina interna	Internal medicine
MED/10	Malattie dell'apparato respiratorio	Respiratory diseases
MED/11	Malattie dell'apparato cardiovascolare	Cardiovascular diseases
MED/12	Gastroenterologia	Gastroenterology
MED/13	Endocrinologia	Endocrinology
MED/14	Nefrologia	Nephrology

MED/15	Malattie del sangue	Blood diseases
MED/16	Reumatologia	Rheumatology
MED/17	Malattie infettive	Infectious diseases
MED/18	Chirurgia generale	General surgery
MED/19	Chirurgia plastica	Plastic surgery
MED/20	Chirurgia pediatrica e infantile	Paediatric surgery
MED/21	Chirurgia toracica	Thoracic surgery
MED/22	Chirurgia vascolare	Vascular surgery
MED/23	Chirurgia cardiaca	Heart surgery
MED/24	Urologia	Urology
MED/25	Psichiatria	Psychiatry
MED/26	Neurologia	Neurology
MED/27	Neurochirurgia	Neurosurgery
MED/28	Malattie odontostomatologiche	Oral diseases and dentistry
MED/29	Chirurgia maxillofacciale	Maxillofacial surgery
MED/30	Malattie apparato visivo	Eye diseases
MED/31	Otorinolaringoiatria	Otorhinolaryngology
MED/32	Audiologia	Audiology
MED/33	Malattie apparato locomotore	Musculoskeletal system diseases
MED/34	Medicina fisica e riabilitativa	Physical and rehabilitation medicine
MED/35	Malattie cutanee e veneree	Dermatological and venerological diseases
MED/36	Diagnostica per immagini e radioterapia	Diagnostic imaging and radiotherapy
MED/37	Neuroradiologia	Neuroradiology
MED/38	Pediatria generale e specialistica	General and subspecialty paediatrics

MED/39	Neuropsichiatria infantile	Child neuropsychiatry
MED/40	Ginecologia e ostetricia	Obstetrics and gynaecology
MED/41	Anestesiologia	Anaesthesiology
MED/42	Igiene generale e applicata	Hygiene and public health
MED/43	Medicina legale	Forensic medicine
MED/44	Medicina del lavoro	Occupational medicine
MED/45	Scienze infermieristiche generali, cliniche e pediatriche	Nursing sciences: general, clinical and paediatric
MED/47	Scienze tecniche di medicina di laboratorio scienze infermieristiche ostetrico-ginecologiche	Medical and biotechnology laboratory techniques
MED/48	Scienze infermieristiche e tecniche neuro-psichiatriche e riabilitative	Midwifery
MED/49	Scienze tecniche dietetiche applicate	Neuropsychiatric and rehabilitation nursing sciences
MED/50	Scienze tecniche mediche applicate	Food sciences and dietetics

3. Results

3.1. Gender disparities in Italian academic Medical Area in 2023

In 2023, the career scissor diagram - one of the most common representations of the phenomenon of vertical segregation in academia - showed a predominance of women among research fellows (71.5% women vs. 28.5% men) and RTDA (57.1% women vs. 42.9% men). However, the curves showed

how the proportion of women gradually decreased in the subsequent career phases, for RTDB (40.8% vs. 59.2), researchers (46.8% vs. 59.2%), associate professors (38.2% vs. 61.8%), and full professors (21.9% vs. 78.1%) (**Figure 1**).

Figure 1. Career scissor diagram in Italian Medical Area in 2023

Interestingly, the gender distribution of Temporary Extraordinary Professors showed a predominance of males (91.1 % vs. 8.9 %; **Figure 2**).

Figure 2. Gender distribution of Temporary Extraordinary Professor in Italian Medical Area in 2023

Gender disparity is prevalent across most SSDs. Specifically, a gender segregation with a clear predominance of men was observed for the following SSDs: MED/10 - Respiratory diseases, MED/11 – Cardiovascular diseases, MED/13 - Endocrinology, MED/14 - Nephrology, MED/18 - General surgery, MED/19 – Plastic surgery, MED/20 – Paediatric surgery, MED/21 – Thoracic surgery, MED/22 – Vascular surgery, MED/23 – Heart surgery, MED/24 - Urology, MED/27 - Neurosurgery, MED/28 - Oral diseases and dentistry, MED/29 - Maxillofacial surgery, MED/30 - Eye diseases, MED/31 - Otorhinolaryngology, MED/33 - Musculoskeletal system diseases, MED/37 - Neuroradiology, MED/40 - Obstetrics and gynaecology, MED/41 - Anaesthesiology. Conversely, there were other fields where gender segregation is less pronounced, and women equal or surpass the percentage of men (e.g., MED/01 - Medical statistics, MED/02 - Medical history, MED/03 - Medical genetics, MED/04 - Experimental medicine and pathophysiology, MED/05 - Clinical pathology, MED/07 - Microbiology and clinical microbiology, MED/38 - General and subspecialty paediatrics, MED/39 - Child neuropsychiatry, MED/42 - Hygiene and public health, MED/45 - Nursing sciences:

general, clinical and paediatric, MED/47 - Medical and biotechnology laboratory techniques, MED/48 - Midwifery, MED/49 - Neuropsychiatric and rehabilitation nursing sciences, MED/50 - Food sciences and dietetics). However, for the SSDs MED/38, MED/39, MED/42, MED/45, MED/49, and MED/50, there continues to be a predominance of men in the role of full professor. Moreover, in general, women were more frequently represented in Research Fellow and RTDA roles but were underrepresented at higher academic levels. However, this pattern does not hold in SSDs MED/22, MED/24, MED/27, MED/33, and MED/37, where men outnumber women in all academic positions. Notably, in SSD MED/20, men held a larger proportion of Research Fellow positions, while women were predominantly found in RTDA roles. Further details are shown in **Table 2**.

Table 2. Gender disparity within SSDs

SSD	Gender	Research Fellows	RTDA	RTDB	Tenured Researches	Associate Professors	Full Professors	Temporary Extraordinary Professor
**MED/01**	F	73.3%	68.8%	50.0%	50.0%	63.8%	51.2%	
	M	26.7%	31.3%	50.0%	50.0%	36.2%	48.8%	
**MED/02**	F	80.0%	66.7%	50.0%	40.0%	46.7%	66.7%	
	M	20.0%	33.3%	50.0%	60.0%	53.3%	33.3%	
**MED/03**	F	83.9%	64.7%	68.4%	82.4%	58.2%	39.6%	
	M	16.1%	35.3%	31.6%	17.6%	41.8%	60.4%	
**MED/04**	F	76.0%	74.7%	63.9%	72.9%	61.9%	40.8%	
	M	24.0%	25.3%	36.1%	27.1%	38.1%	59.2%	100.0%
**MED/05**	F	85.0%	88.9%	72.7%	73.3%	70.5%	46.7%	
	M	15.0%	11.1%	27.3%	26.7%	29.5%	53.3%	
**MED/06**	F	76.7%	50.0%	40.0%	50.0%	30.6%	15.0%	33.3%
	M	23.3%	50.0%	60.0%	50.0%	69.4%	85.0%	66.7%
**MED/07**	F	69.4%	76.3%	55.6%	63.6%	71.6%	50.0%	
	M	30.6%	23.7%	44.4%	36.4%	28.4%	50.0%	
**MED/08**	F	63.3%	50.0%	52.4%	57.7%	44.9%	32.0%	
	M	36.7%	50.0%	47.6%	42.3%	55.1%	68.0%	
**MED/09**	F	68.4%	48.3%	36.2%	51.4%	33.9%	16.9%	
	M	31.6%	51.7%	63.8%	48.6%	66.1%	83.1%	100.0%
**MED/10**	F	81.0%	64.3%	30.0%	37.5%	39.5%	19.4%	
	M	19.0%	35.7%	70.0%	62.5%	60.5%	80.6%	100.0%
**MED/11**	F	76.3%	54.8%	19.4%	35.5%	29.7%	8.2%	
	M	23.7%	45.2%	80.6%	64.5%	70.3%	91.8%	100.0%
**MED/12**	F	76.7%	45.5%	50.0%	71.4%	26.6%	19.0%	

	M	23.3%	54.5%	50.0%	28.6%	73.4%	81.0%	100.0%
**MED/13**	F	87.1%	60.0%	63.6%	35.7%	48.5%	19.0%	
	M	12.9%	40.0%	36.4%	64.3%	51.5%	81.0%	
**MED/14**	F	83.3%	47.1%	50.0%	50.0%	27.3%	14.3%	50.0%
	M	16.7%	52.9%	50.0%	50.0%	72.7%	85.7%	50.0%
**MED/15**	F	67.6%	69.0%	36.4%	52.6%	41.0%	12.5%	
	M	32.4%	31.0%	63.6%	47.4%	59.0%	87.5%	100.0%
**MED/16**	F	80.0%	61.5%	30.0%	66.7%	54.4%	19.2%	
	M	20.0%	38.5%	70.0%	33.3%	45.6%	80.8%	
**MED/17**	F	77.8%	50.0%	38.9%	56.0%	37.9%	25.0%	
	M	22.2%	50.0%	61.1%	44.0%	62.1%	75.0%	100.0%
**MED/18**	F	76.2%	30.4%	23.9%	23.1%	11.3%	3.5%	
	M	23.8%	69.6%	76.1%	76.9%	88.7%	96.5%	100.0%
**MED/19**	F	66.7%	40.0%	26.7%	20.0%	12.5%	3.8%	
	M	33.3%	60.0%	73.3%	80.0%	87.5%	96.2%	100.0%
**MED/20**	F	33.3%	66.7%	33.3%	40.0%	9.5%	14.3%	
	M	66.7%	33.3%	66.7%	60.0%	90.5%	85.7%	
**MED/21**	F	100.0%	50.0%	50.0%	0%	7.7%	5.3%	
	M	0%	50.0%	50.0%	100.0%	92.3%	94.7%	
**MED/22**	F	0%	44.4%	0%	18.2%	12.2%	0%	
	M	100.0%	55.6%	100.0%	81.8%	87.8%	100.0%	
**MED/23**	F	66.7%	57.1%	0%	0%	2.4%	3.6%	
	M	33.3%	42.9%	100.0%	100.0%	97.6%	96.4%	
**MED/24**	F	40.0%	7.1%	6.7%	0%	2.1%	2.2%	
	M	60.0%	92.9%	93.3%	100.0%	97.9%	97.8%	
**MED/25**	F	60.6%	40.9%	38.5%	37.5%	43.1%	24.4%	
	M	39.4%	59.1%	61.5%	62.5%	56.9%	75.6%	100.0%

**MED/26**	F	64.0%	47.0%	39.5%	50.0%	41.1%	17.4%	100.0%
	M	36.0%	53.0%	60.5%	50.0%	58.9%	82.6%	
**MED/27**	F	37.5%	20.0%	12.5%	16.7%	5.1%	3.1%	
	M	62.5%	80.0%	87.5%	83.3%	94.9%	96.9%	100.0%
**MED/28**	F	60.4%	31.3%	31.7%	36.8%	22.2%	21.5%	
	M	39.6%	68.8%	68.3%	63.2%	77.8%	78.5%	100.0%
**MED/29**	F	50.0%	14.3%	22.2%	18.2%	14.3%	0%	
	M	50.0%	85.7%	77.8%	81.8%	85.7%	100.0%	100.0%
**MED/30**	F	56.0%	41.4%	53.8%	39.0%	24.7%	4.7%	
	M	44.0%	58.6%	46.2%	61.0%	75.3%	95.3%	100.0%
**MED/31**	F	90.9%	22.2%	17.6%	23.5%	13.6%	0%	
	M	9.1%	77.8%	82.4%	76.5%	86.4%	100.0%	100.0%
**MED/32**	F	75.0%	16.7%	100.0%	62.5%	36.7%	37.5%	
	M	25.0%	83.3%	0%	37.5%	63.3%	62.5%	
**MED/33**	F	33.3%	22.2%	3.2%	11.1%	9.3%	0%	
	M	66.7%	77.8%	96.8%	88.9%	90.7%	100.0%	100.0%
**MED/34**	F	43.8%	54.5%	40.0%	57.1%	35.1%	25.9%	
	M	56.3%	45.5%	60.0%	42.9%	64.9%	74.1%	
**MED/35**	F	65.6%	62.5%	68.2%	64.3%	46.6%	32.3%	
	M	34.4%	37.5%	31.8%	35.7%	53.4%	67.7%	100.0%
**MED/36**	F	62.2%	35.9%	24.4%	50.0%	35.7%	16.3%	100.0%
	M	37.8%	64.1%	75.6%	50.0%	64.3%	83.7%	
**MED/37**	F	0%	20.0%	10.0%	0%	31.6%	21.1%	
	M	100.0%	80.0%	90.0%	100.0%	68.4%	78.9%	100.0%
**MED/38**	F	76.4%	73.8%	51.4%	58.8%	51.6%	23.3%	50.0%
	M	23.6%	26.2%	48.6%	41.2%	48.4%	76.7%	50.0%
**MED/39**	F	78.9%	91.7%	68.8%	72.7%	64.9%	30.0%	

	M	21.1%	8.3%	31.3%	27.3%	35.1%	70.0%	
**MED/40**	F	88.9%	46.2%	44.8%	63.4%	38.1%	13.1%	
	M	11.1%	53.8%	55.2%	36.6%	61.9%	86.9%	100.0%
**MED/41**	F	53.3%	41.7%	30.0%	45.9%	23.9%	12.5%	
	M	46.7%	58.3%	70.0%	54.1%	76.1%	87.5%	100.0%
**MED/42**	F	75.0%	63.0%	53.6%	65.0%	63.2%	36.8%	
	M	25.0%	37.0%	46.4%	35.0%	36.8%	63.2%	100.0%
**MED/43**	F	76.5%	35.3%	36.8%	40.0%	37.8%	23.3%	
	M	23.5%	64.7%	63.2%	60.0%	62.2%	76.7%	100.0%
**MED/44**	F	52.0%	53.3%	35.7%	60.0%	44.4%	25.0%	
	M	48.0%	46.7%	64.3%	40.0%	55.6%	75.0%	100.0%
**MED/45**	F	88.9%	57.9%	28.6%		56.7%	75.0%	
	M	11.1%	42.1%	71.4%		43.3%	25.0%	
**MED/46**	F	76.4%	87.7%	64.3%	70.0%	70.1%	52.8%	
	M	23.6%	12.3%	35.7%	30.0%	29.9%	47.2%	
**MED/47**	F		100.0%		100.0%	100.0%		
	M		0.0%		0.0%	0.0%		
**MED/48**	F	55.6%	70.0%	10.0%	33.3%	73.3%	57.1%	
	M	44.4%	30.0%	90.0%	66.7%	26.7%	42.9%	
**MED/49**	F	77.1%	73.7%	77.3%	45.5%	52.5%	34.6%	
	M	22.9%	26.3%	22.7%	54.5%	47.5%	65.4%	
**MED/50**	F	64.1%	79.2%	53.6%	45.5%	45.5%	28.9%	
	M	35.9%	20.8%	46.4%	54.5%	54.5%	71.1%	100.0%

3.2.Gender disparities across Italian regions in 2023

We also analysed gender disparity across Italian regions using the GCI and GDI. Our findings indicate a presence of a glass ceiling effect in all Italian regions, suggesting that women are underrepresented

in Full Professor positions compared to other academic roles. Excluding regions with incomplete data (i.e., Basilicata, Molise, and Valle d'Aosta), Sardinia, Liguria, and Veneto displayed the highest levels of gender inequality, disadvantaging women, with GCI values of 2.9, 2.5, and 2.3, respectively. Conversely, the lowest GCI values were observed in Piedmont (1.4), Trentino-Alto Adige (1.3), and Friuli-Venezia Giulia (1.2) (**Figure 3**).

Figure 3. Gender disparity in Italian Medical Area in 2023 according to Glass Ceiling Index

According to the GDI, only the regions of Molise and Trentino-Alto Adige reported a value below one, highlighting that in the tenure track position, the percentage of women is higher (or equal) compared to the percentage of women in temporary positions. This result must be considered cautiously as it could be only due to the low number of professionals in the academic roles in these regions. By contrast, the highest GDI values were detected in Abruzzo (2.7), Apulia (2.2), Sardinia (2.1) (**Figure 4**).

Figure 4. Gender disparity in Italian Medical Area in 2023 according to Glass Door Index

3.3. Gender disparities in Italian academic Medical Area from 2014 to 2023

We next assessed the temporal trend of female-to-male ratio from 2015 to 2023, according to academic roles (**Figure 5**). Over time, there has been a gradual rise in female-to-male ratios, suggesting an increasing presence of women across all academic positions. This increase has been moderate across most roles, apart from RTDA positions, where a more significant rise has been observed. It is worthwhile to note that gender parity (a female-to-male ratio of 1) has not been achieved, except in the RTDA category.

Figure 5. Temporal trend of female-to-male ratio from 2015 to 2023

We next assessed the temporal trend - from 2015 to 2023 – of gender disparities in Italian academic Medical Area. According to GCI, the temporal analysis showed a reduction in female disparities in academic top positions, from 2.3 in 2015 to 1.7 in 2023. Moreover, GDI increased from 1.3 in 2015 to 1.6 in 2023 (**Figure 6**).

Figure 6. Temporal trends of gender disparities from 2015 to 2023 according to GCI and GDI

We also examined temporal trends in gender disparities within regions using GDI and GCI.

In general, the GCI remained above 1 for all regions and for all the years considered, with a tendency toward a reduction of vertical segregation. Specifically, Friuli-Venezia Giulia and Veneto showed a more pronounced improvement (from 4.0 in 2014 to 1.2 in 2023, and from 3.7 to 2.3 in 2023, respectively). The only exception has been Sardinia, where there was a worsening trend toward vertical segregation (from 1.7 in 2014 to 2.9 in 2023).

The GDI values, on the other hand, have exhibited heterogeneous changes from 2014 to 2023, with most cases showing an increase in disparity to the detriment of the female gender in 2023.

Caution is necessary when interpreting the indices for regions with a low number of professionals in various academic roles (e.g., Molise and Trentino-Alto Adige). Further details can be found in **Figure 7** and **Figure 8**.

Figure 7. Temporal trends of gender disparities within Regions according to GCI

Figure 8. Temporal trends of gender disparities within Regions according to GDI

Next, we assessed temporal trends of gender disparities within SSDs according to GDI and GCI. In general, the GCI usually reported a value greater than one, with some exceptions: SSD MED/02 from 2021 to 2023, MED/21 from 2014 to 2016, MED/23 in 2016 and from 2018 to 2020, MED/45 from 2014 to 2023, MED/47 in 2017, and MED/48 in 2014-2015. Considering the GDI, the situation was more heterogeneous, and results should be analysed SSD by SSD. Notably, SSD MED/45, which already showed a good percentage of women in various roles (see paragraph 3.1), reported a GCI of 0.7 in 2023—stable compared to 2014—and a GDI of 2.0, up from 0.3 in 2015. Further details can be found in **Figure 9 and Figure 10.**

Figure 9. Temporal trends of gender disparities within SSD according to GCI

Figure 10. Temporal trends of gender disparities within SSD according to GDI

4. Discussion

Discrimination against women in the workplace remains a significant challenge, despite ongoing efforts to promote gender equality. According to the European Institute for Gender Equality (EIGE), gender segregation and discrimination persist across various professional fields, with women facing systemic barriers to career advancement and leadership positions [1]. The "Gender Equality Index 2020" report by EIGE highlights that women are underrepresented in decision-making roles and experience a substantial pay gap compared to their male counterparts. This disparity is even more pronounced in sectors such as STEM, where women tend to be concentrated in lower-paying, junior roles, while senior leadership roles remain predominantly male [3]. Additionally, the European Commission's "She Figures 2021" report reveals that women are disproportionately affected by precarious employment conditions and have limited access to research funding and career development opportunities in academia [4]. Scientific evidence further underscores these disparities

[18] highlighting the multifaceted nature of these disparities, which manifest in different forms across academic disciplines and career stages [19-23]. Research also indicates that gender disparities are not uniform across disciplines. However, even in more gender-balanced fields, women tend to face significant barriers when it comes to attaining senior academic positions and receiving recognition for their work [24]. Moreover, research by the European Commission demonstrates that unconscious biases and structural barriers, such as the lack of family-friendly policies and support for work-life balance, contribute to the ongoing discrimination against women in the workplace.

The use of the GCI and GDI has allowed us to assess gender disparities in the Italian academic medical field. Specifically, in the context of the Italian academic medical field, these indices serve as valuable tools for identifying barriers that women face in reaching leadership roles and their participation in high-ranking academic positions. The GCI measures the extent to which women face obstacles in advancing to top leadership positions, reflecting the metaphorical "ceiling" that limits their progression. In the context of Italian academia, studies have demonstrated that while women may achieve significant representation at lower academic levels, they tend to be underrepresented in senior roles such as professors, department heads, and deans. The GCI helps quantify this disparity, indicating how the progression of women in academia slows down as they move up the academic hierarchy. On the other hand, the GDI assesses the barriers women encounter when attempting to enter academic fields or institutions, essentially evaluating how open or closed the "door" is to women at the onset of their academic careers. It looks at factors like recruitment practices, access to research funding, and the gendered dynamics of hiring decisions. In the Italian medical academic sector, evidence from the GDI shows that while women are well-represented in medical education, their participation drops in higher positions, which is influenced by unconscious bias, gendered perceptions of leadership, and institutional structures that favour me[illegible] In Italy, these indices have been instrumental in demonstrating that despite various initiatives for gender equality, there remain significant gender gaps in medical academia. The gender imbalances are not only seen in leadership

roles but also in areas like research funding allocation and career advancement opportunities. By using the GCI and GDI, these studies highlight the need for policies that promote gender equality at all stages of an academic career—ensuring that women are not only able to enter the field but also have the support and opportunities to thrive and lead in their respective fields. Incorporating these tools into research also opens the door for more targeted interventions. For example, using the GCI and GDI can guide institutions to implement specific gender equality measures, such as mentorship programs, family-friendly policies, and transparent recruitment processes, that aim to dismantle the structural and cultural barriers that contribute to gender disparity in academic medicine. These indices provide a clear and quantifiable way to track progress and design more effective strategies to foster gender equity within the medical academic sector. The aim was to test the hypothesis that, also in this area, the gender gap in academic careers is not going to disappear in a relatively short time. As it has been shown by recent research findings the issue is not just that of waiting the time needed to see the new generations of women that have entered academia break the glass ceiling. The gender gap is self-sustaining through different mechanisms at different stages of the academic career, as the glass door effect revealed is happening at the transition from temporary positions to the tenure track position, after its recent introduction in the Italian university system [7, 17, 25, 26] by the latest general reform of the Italian university system (Law 240/2010, better known as the Gelmini Reform). Our findings confirm the hypothesis for the medical area, showing that a higher number of women in early career stages does not correspond to a proportionate rate of women in tenure track positions. By contrast, as the percentage of women in the initial stages increases, the presence of obstacles to stabilization becomes more evident. Despite regulatory interventions and gender mainstreaming policies, the career scissor diagram for 2023 clearly illustrates the permanence of the decline in the representation of women as they advance through academic ranks, culminating in a significant underrepresentation at the full professor level. The temporal trend analysis from 2014 to 2023 offers a glimmer of hope, revealing a gradual increase in the female-to-male ratio across academic roles and a reduction in gender disparities in leadership positions. However, the pace of change remains slow, and achieving

gender parity is still a distant goal for most academic roles. Furthermore, as Ferree and Purkayastha suggest, the decrease of a disadvantage in the transition from the intermediate (B) to the final level (A), is not unambiguously signalling greater gender equality to the extent that, on the one hand, women who are at the first level are still less likely to become tenured researchers than their male colleagues and, on the other hand, this decrease could be due precisely to the discrimination that women experience in the previous step [27].

Through the introduction of the GDI, the analysis empirically highlights the existence of gender differentiation processes in academic career paths within the medical area, starting from the recruitment phases. Specifically, the career scissor diagram varies significantly across different disciplines, and the comparison between GDI values before the reform and after the reform shows that, following the Gelmini reform, there has been a systematic relative decrease in the number of women accessing stable academic positions [17]. Comparison between SSDs has revealed disciplines that historically show a predominance of the male gender, ~~as~~ especially surgical specialties. The higher percentage of men in these sectors may be attributed to various historical, cultural, and social factors. Traditionally, many of these fields have been perceived as more technical or less compatible with roles historically assigned to women. This may have led to lower initial participation of women and, consequently, to a persistence of gender disparity. Additionally, structural barriers and gender bias, the lack of female role models, and traditional gender expectations as well as the shortage in services regarding family responsibilities may have contributed to discouraging them from pursuing or continuing careers in these fields. Conversely, other disciplines, such as nursing sciences, show a predominance of women, although horizontal segregation persists.

The observed trend is consistent with broader European patterns, where gender disparities in academia are well-documented. The observed predominance of women in early career stages, such as research fellows and RTDA, contrasts sharply with their dwindling presence in senior positions, underscoring the persistence of structural barriers and potentially unconscious biases that hinder

women's career progression. As noted in several European studies, women are often underrepresented in higher-ranking positions, despite their significant contributions in early career stages [4]. For example, in the "She Figures 2021" report, women account for just 29% of full professors in Europe, despite representing nearly half of all doctoral graduates and postdoctoral researchers [4]. The predominance of women in the early career stages, particularly in research-focused roles, may be partly attributed to factors such as greater flexibility in these positions, which are sometimes more accessible for individuals balancing work and family responsibilities. However, as careers progress, the proportion of women drops substantially in response to institutional and cultural barriers. Women’s advancement is often slowed by the so-called "leaky pipeline," a phenomenon where women enter academia in equal or greater numbers than men but face increasing barriers that prevent them from reaching senior leadership roles. This includes not only biased career evaluation systems but also the challenges posed by work-life balance issues, insufficient mentorship, and unequal access to professional networks, which are often critical for career advancement [12]. Geographical disparities further compound this issue, with regions like Sardinia, Liguria, and Veneto exhibiting the highest gender inequalities. In contrast, regions such as Piedmont and Trentino-Alto Adige show more equitable distributions, indicating regional variations in the effectiveness of gender equality policies. The GCI and GDI reinforce the notion that systemic barriers remain prevalent, with few regions demonstrating substantial progress in achieving gender parity in recruitment and promotion processes. The temporal trend analysis from 2014 to 2023 offers a glimmer of hope, revealing a gradual increase in the female-to-male ratio across academic roles and a reduction in gender disparities in leadership positions. However, the pace of change remains slow, and achieving gender parity is still a distant goal for most academic roles. As Ferree and Purkayastha suggest, the decrease of a disadvantage between adjacent levels, and more particularly in the transition from the intermediate (B) to the final level (A), does not necessarily mean greater gender equality to the extent that, on the one hand, women who are at the first level are still less likely to become tenured researchers than their male colleagues and, on the other hand, this decrease could be due precisely to

the discrimination that women experience in the previous step [27]. The regional distribution of inequalities also suggests possible hypotheses about the gender distribution: women may be overrepresented in the highest positions in regions that are less desirable for logistic and prestige reasons as the case of Molise (a mountainous region with logistic difficulties, especially during the winter season) may suggest – although small numbers in the data call for cautious interpretations.

The study has also several limitations. The lack of data on the number of research fellows in the years preceding 2023. As a result, this category has been excluded from the calculation of the GCI and GDI, potentially leading to an incomplete picture of gender inequality in early career stages. Moreover, the emphasis on quantitative data might overlook qualitative aspects of gender discrimination, such as personal experiences and organizational cultures that also play significant roles in shaping women's career trajectories through context specific social mechanisms [28]. The study highlights that gender disparities are not uniform across disciplines. However, the research could benefit from a more detailed analysis of why certain fields are more gender-balanced than others, which might offer insights into more effective gender equality strategies. While the study mentions unconscious biases and structural barriers and suggests possible contextual mechanisms being at work in different regions, further research could delve deeper into these aspects to offer more concrete recommendations on how to address them [28].

5. Conclusion

In conclusion, while there have been notable advancements in gender equality in the Italian academic medical field, persistent inequalities continue to hinder the full integration and progression of women in the sector. These inequalities are evident not only in the underrepresentation of women in leadership roles, such as senior professors, heads of departments, and academic deans, but also in the unequal distribution of research funding and access to career advancement opportunities. Despite various initiatives aimed at promoting gender equality, these disparities remain entrenched due to

both visible and invisible structural barriers that continue to favour male counterparts. Our findings suggest that while the glass ceiling remains a significant issue, there is also a pressing need to address the *glass door* phenomenon—the barriers preventing women from entering academic medicine in the first place. Factors such as unconscious bias in recruitment practices, the prioritization of male-dominated networks, and societal expectations surrounding gender roles contribute to this uneven playing field. These issues require a multi-faceted response that targets both the entry-level barriers and the challenges faced by women as they progress through their academic careers. More robust and targeted strategies are necessary to dismantle these structural barriers. Institutional policies should focus not only on ensuring fair and equitable recruitment practices but also on promoting transparent and inclusive promotion processes. This involves revising performance evaluation metrics that traditionally favour male-dominated research areas and ensuring that caregiving responsibilities, which disproportionately fall on women, are not seen as obstacles to career progression. Mentorship programs, networking opportunities, and leadership training tailored specifically for women can also serve as crucial steps in addressing the systemic biases that persist in the field. Furthermore, fostering an inclusive academic environment requires cultural shifts within academic institutions. This includes promoting work-life balance policies, supporting parental leave, and creating family-friendly working conditions to reduce the gendered expectations placed on women. These cultural changes should be coupled with comprehensive policy implementation and sustained monitoring to track progress toward achieving gender equality. The road to achieving lasting gender equality in Italian academic medicine is undeniably challenging, as it requires overcoming deeply rooted biases, norms, and practices. However, it is a crucial goal that will not only benefit women but also enrich the academic medical community as a whole. Studies consistently show that diversity in leadership fosters better decision-making, more innovative research, and improved outcomes across various sectors, including healthcare. Therefore, addressing gender inequality is not just a matter of social justice but also an imperative for improving the quality of education and research in the medical field. Achieving true gender equality requires continuous, multi-dimensional efforts that include policy reforms, cultural

transformation, and institutional support. The success of these efforts will depend on the willingness of institutions to recognize and address the systemic biases that perpetuate inequality, ultimately creating a more inclusive and equitable environment that allows all individuals, regardless of gender, to reach their full potential in academia.

Acknowledgments

The authors thank editor and our anonymous reviewers for their important feedback and guidance. The authors also thank the Gender Prevention Working Group of the Italian Society of Hygiene, Preventive Medicine, and Public Health - SItI.

References

1. European Institute for Gender Equality, EIGE. Gender Equality in Academia and Research GEAR tool step. 2022. Available at: https://eige.europa.eu/sites/default/files/documents/20220795_pdf_mh0922276enn_002.pdfby-step guide.

2. Farina, S., Iafrate, R., and Boccia, S. 2023. "Unveiling the gender gap: exploring gender disparities in European academic landscape." Lancet Reg Health Eur 34:100752. doi: 10.1016/j.lanepe.2023.100752.

3. European Institute for Gender Equality, EIGE. Gender Equality Index 2020: Key Findings for the EU. Available at: https://eige.europa.eu/sites/default/files/documents/20203354_mhag20029enn_002.pdf

4. European Commission, 2021. She Figures 2021, Gender in research and innovation: statistics and indicators. Available at: https://op.europa.eu/it/publication-detail/-/publication/67d5a207-4da1-11ec-91ac-01aa75ed71a15.

5. Liccardo A., Agodi MC., Picardi I, Pisanti O., Masullo MR and Gargano A. 2016. Primo bilancio di genere dell'Ateneo federiciano, Napoli, FedOAPress.

6. Celletti A., Costantini P, Primeri E, Romagnosi S. I focus del rapporto ANVUR 2023. Analisi di genere. 2023

7. Murgia A, Poggio B. 2018. Gender and Precarious Research Careers: A Comparative Analysis. https://doi.org/10.4324/9781315201245

8. Picardi I., 2016. Per una dimensione di genere nella valutazione della ricerca, in Avveduto S. et al. (a cura di), Scienza, genere e società. 2016. Prospettive di genere in una società che si evolve, IRPPS Monografie.

9. Picardi I, Agodi M.C, 2017. Il mentoring come strumento di de-costruzione delle strutture di genere riprodotte nell'università e la ricerca, in Murgia A. e Poggio B. (a cura di), Prospettive interdisciplinari su formazione, università, lavoro. 2017.

10. Agodi, M.C, and Picardi I. 2016. "Il mentoring come progetto trasformativo della struttura di genere dell'accademia: il programma pilota di GENOVATE@UNINA."

11. Consorzio Interuniversitario Almalaurea. FOCUS GENDER GAP 2023. Available at: https://www.almalaurea.it/sites/default/files/2023-03/FOCUS-GENDER-GAP_2023.pdf

12. European Commission, 2020. A Union of Equality: Gender Equality Strategy 2020-2025. Available at: https://eur-lex.europa.eu/legal-content/EN/TXT/PDF/?uri=CELEX:52020DC0152

13. Herschberg C., Benschop Y. e van den Brink M. 2018. Precarious postdocs: A comparative study on recruitment and selection of early-career researchers.

14. Ministero dell'Istruzione e del Merito. Integrazione del decreto del Ministro dell'istruzione, dell'università e della ricerca 1 settembre 2016, n. 662, recante la tabella di corrispondenza tra posizioni accademiche italiane ed estere ai sensi dell'articolo 18, comma 1, lettera b), della Legge 30 dicembre 2010, n. 240. Available at: https://www.miur.gov.it/-/integrazione-del-decreto-del-ministro-dell-istruzione-dell-universita-e-della-ricerca-1-settembre-2016-n-662-recante-la-tabella-di-corrispondenza-tra

15. Ministero dell'Istruzione e del Merito. Reclutamento delle Università. Available at: https://www.miur.gov.it/reclutamento-nelle-universita#:~:text=Ricercatori%20a%20tempo%20indeterminato%3A,3%20della%20Legge%20210%2F1998.

16. European Commission, 2015. She Figures 2015. Gender in research and innovation: statistics and indicators. Available at: https://op.europa.eu/it/publication-detail/-/publication/67d5a207-4da1-11ec-91ac-01aa75ed71a1

17. Picardi I. The Glass Door Index: revealing the gendered impact of the Gelmini reform on the access to the academic profession. 2019. Soc. Sci. 2019, 8, 160. https://doi.org/10.3390/socsci8050160

18. Moss-Racusin CA, Dovidio JF, Brescoll VL, Graham MJ, Handelsman J. Science faculty's subtle gender biases favor male students. Proc Natl Acad Sci U S A. 2012;109(41):16474-9. Epub 20120917. doi: 10.1073/pnas.1211286109. PubMed PMID: 22988126; PubMed Central PMCID: PMCPMC3478626.

19. Llorens, A., A. Tzovara, L. Bellier, I. Bhaya-Grossman, A. Bidet-Caulet, W. K. Chang, Z. R. Cross, R. Dominguez-Faus, A. Flinker, Y. Fonken, M. A. Gorenstein, C. Holdgraf, C. W. Hoy, M. V. Ivanova, R. T. Jimenez, S. Jun, J. W. Y. Kam, C. Kidd, E. Marcelle, D. Marciano, S. Martin, N. E.

20. Lombardo E., Naldini M., Poggio B. Gender Inequalities in Academia: Multiple Approaches to Closing the Gap. 2024. https://doi.org/10.3280/sl2024-168001

21. Galvin DJ, Anderson SC, Marolf CJ, Schneider NG, Liebl AL. Comparative analysis of gender disparity in academic positions based on U.S. region and STEM discipline. PLoS One. 2024;19(3):e0298736. Epub 20240320. doi: 10.1371/journal.pone.0298736. PubMed PMID: 38507318; PubMed Central PMCID: PMCPMC10954166.

22. Bendels MHK, Müller R, Brueggmann D, Groneberg DA. Gender disparities in high-quality research revealed by Nature Index journals. PLoS One. 2018;13(1):e0189136. Epub 20180102. doi: 10.1371/journal.pone.0189136. PubMed PMID: 29293499; PubMed Central PMCID: PMCPMC5749692.

23. Ross, M. B., B. M. Glennon, R. Murciano-Goroff, E. G. Berkes, B. A. Weinberg, and J. I. Lane. 2023. "Author Correction: Women are credited less in science than men." Nature 621 (7979):E41. doi: 10.1038/s41586-023-06571-x.

24. Sá C, Cowley S, Shahrin B, Stevenson C, Su A. Disciplinary gender balance, research productivity, and recognition of men and women in academia. PLoS One. 2023;18(12):e0293080. Epub 20231214. doi: 10.1371/journal.pone.0293080. PubMed PMID: 38096215; PubMed Central PMCID: PMCPMC10720991.

25. Gaiaschi, C. and Musumeci, R. (2020). Just a Matter of Time? Women's Career Advancement in Neo-Liberal Academia. An Analysis of Recruitment Trends in Italian Universities, Social Sciences, 9(9): 163. https://doi.org/10.3390/socsci9090163

26. Gaiaschi, Camilla. Doppio Standard: Donne e carriere scientifiche nell'Italia contemporanea (p.414). 2022. Carocci editore S.p.A.. Edizione del Kindle.

27. Ferree MM, Purkayastha B (2000), Equality and Cumulative Disadvantage: Response to Baxter and Wright, in “Gender and Society”, 14, 6, pp. 809-13.

28. Picardi, I. (2020) Labirinti di cristallo, Strutture di genere nell'accademia e nella ricerca, Milano, Franco Angeli

Figure 1

Click here to access/download;Figure;Figure 1.tiff

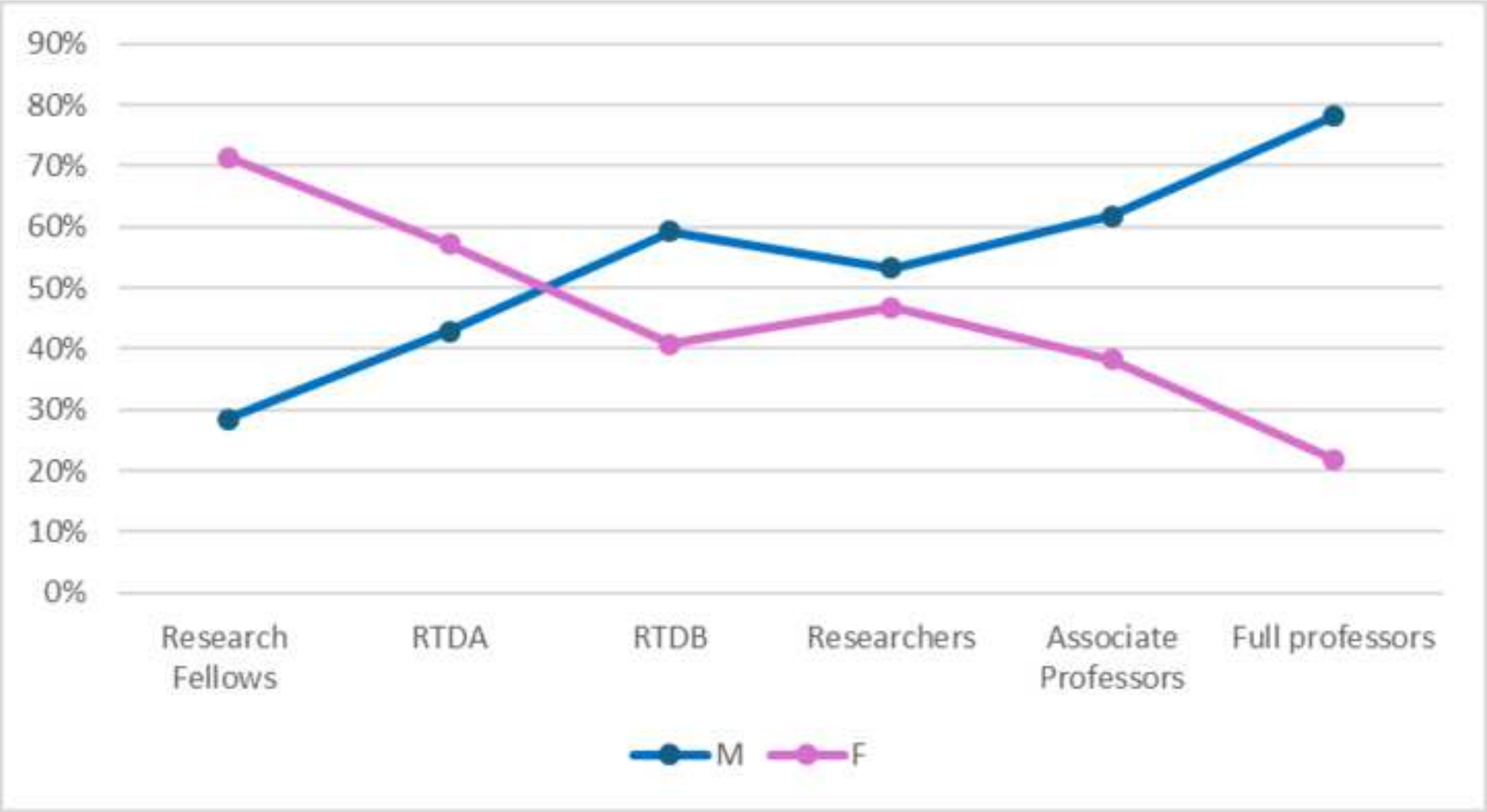

Figure 2

Click here to access/download;Figure;Figure 2.tiff

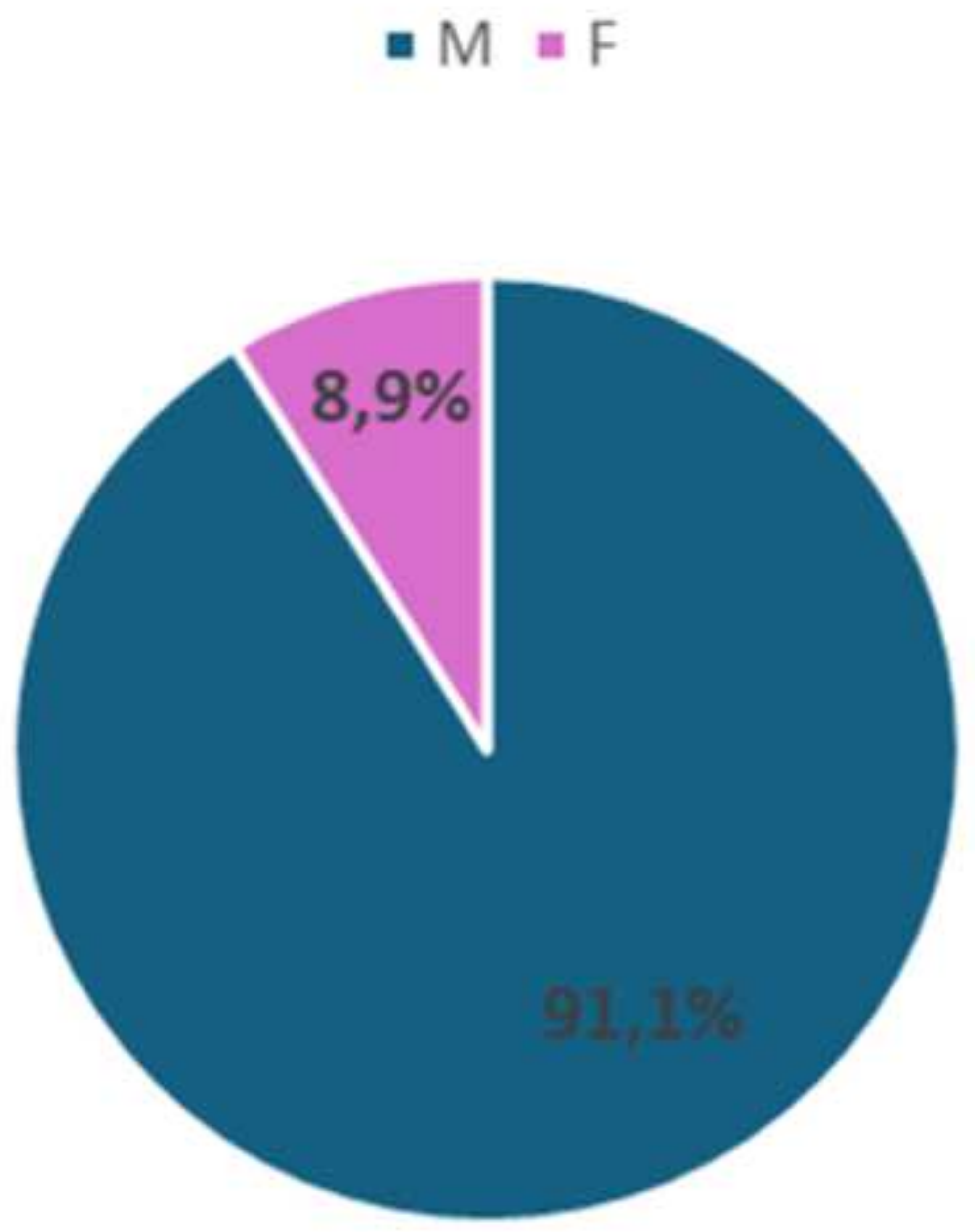

Figure 3

Click here to access/download;Figure;Figure 3.tiff

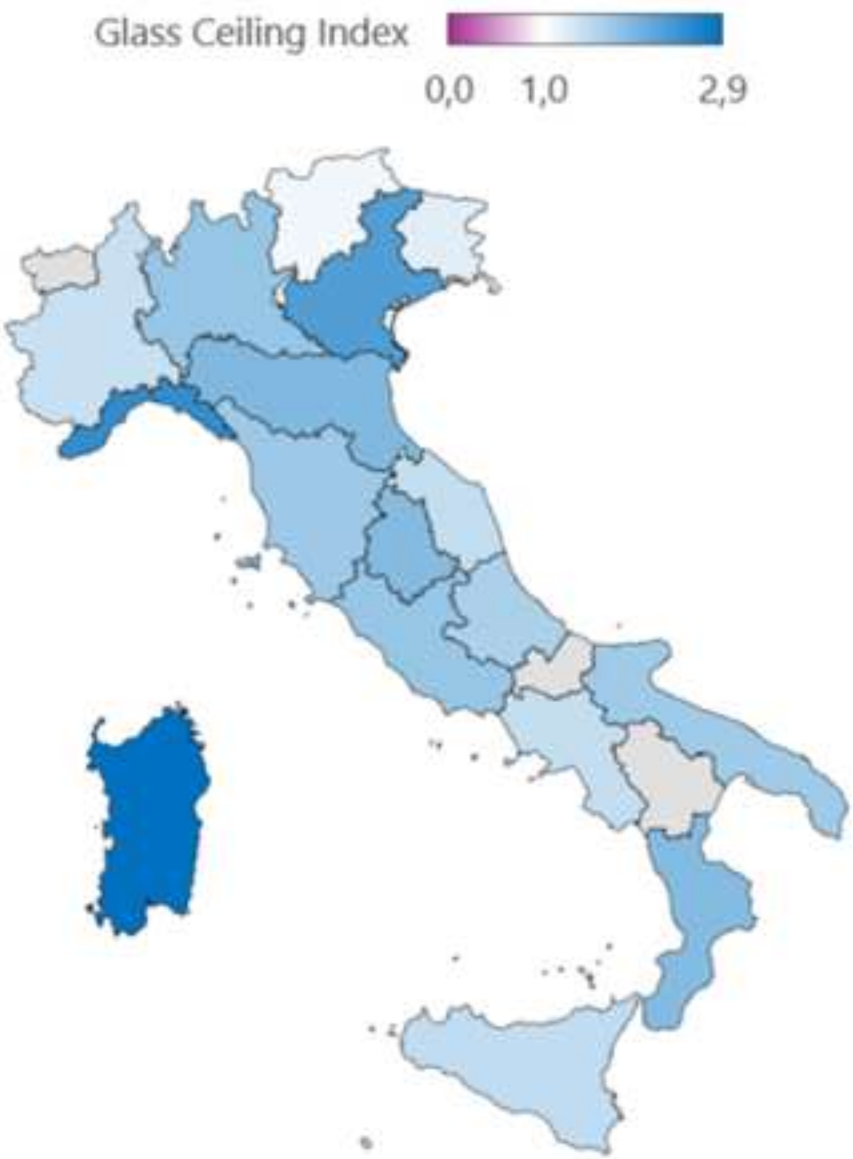

Click here to access/download;Figure;Figure 4.tiff

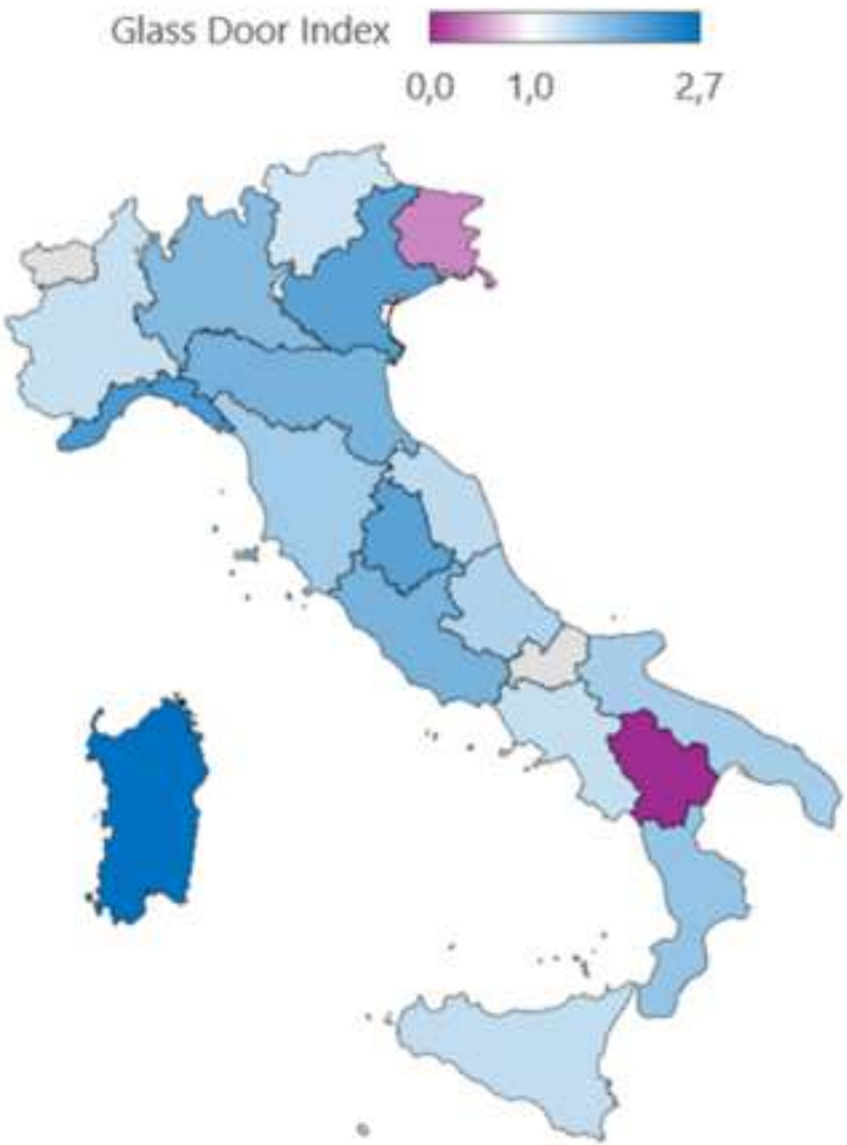

Figure 5

Click here to access/download;Figure;Figure 5.tiff

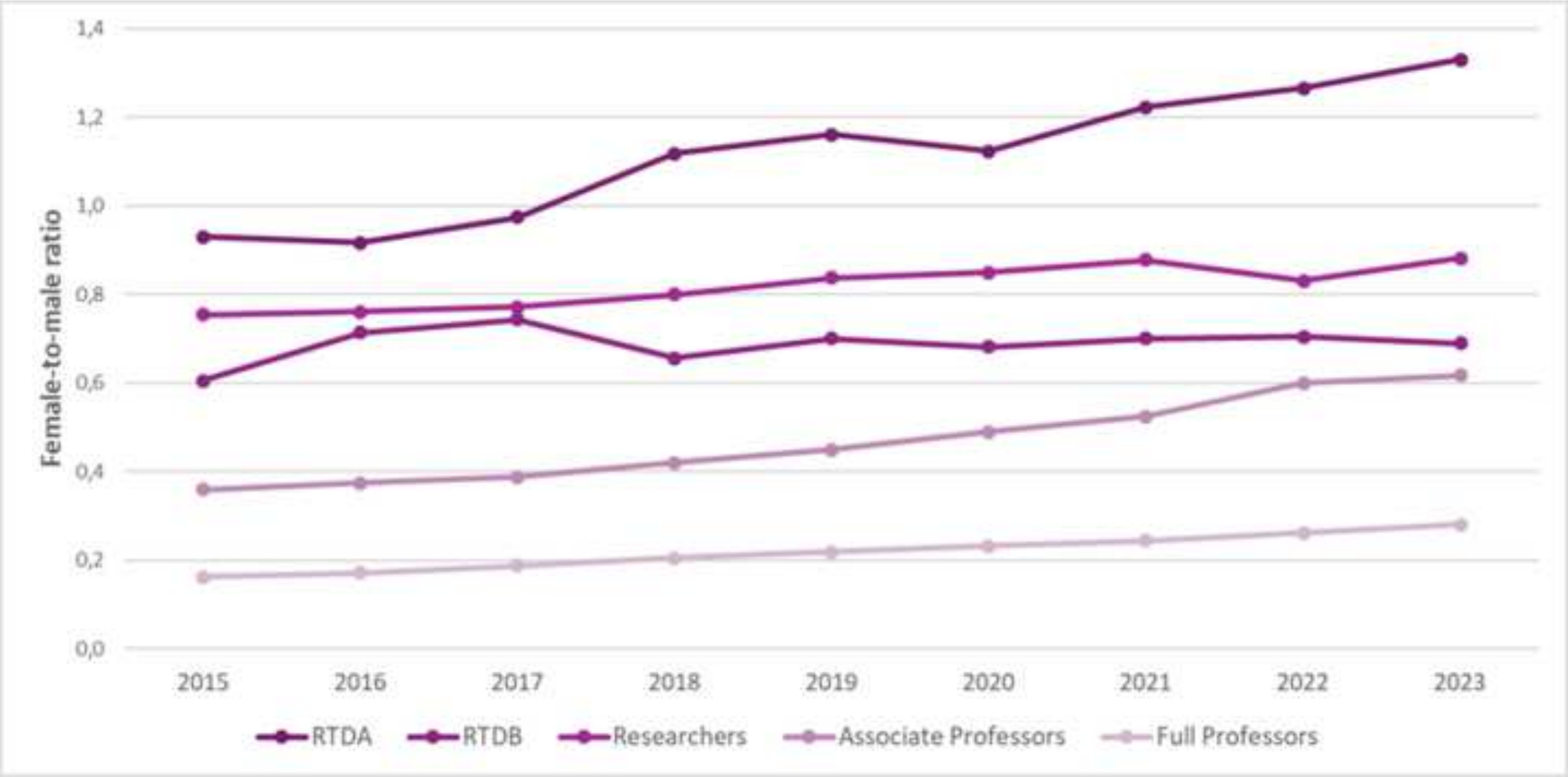

Figure 6

Click here to access/download;Figure;Figure 6.tiff

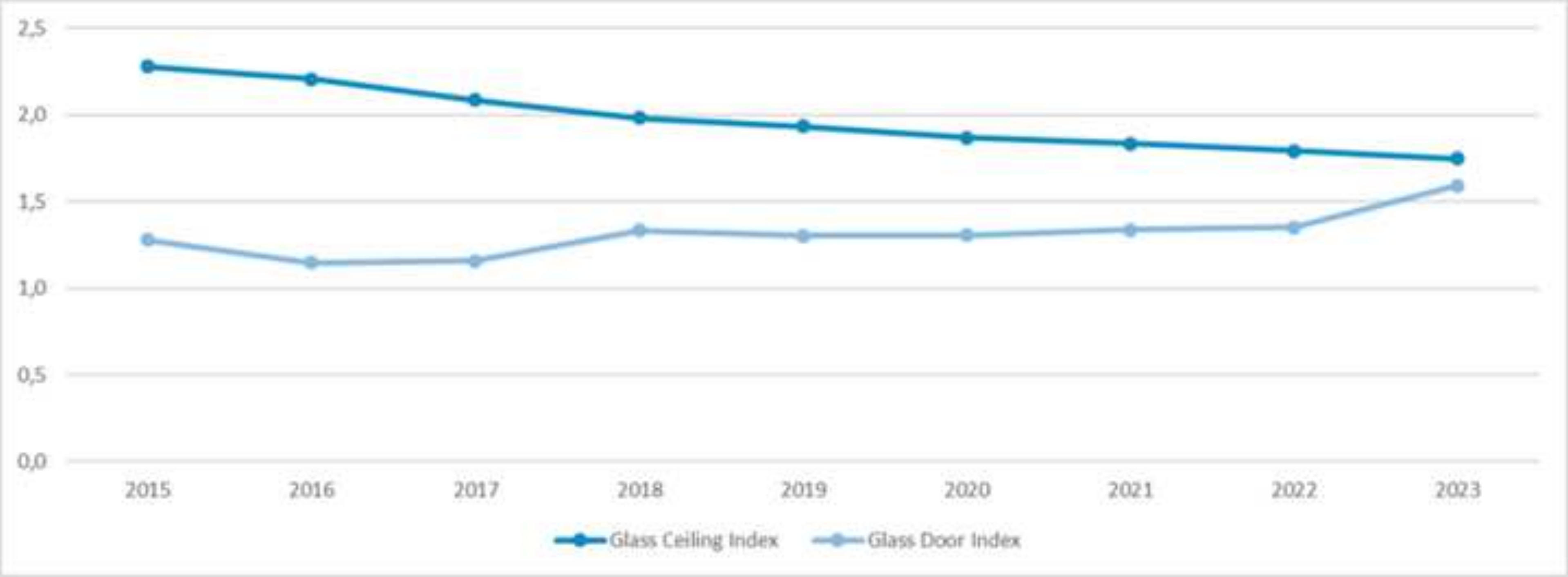

Click here to access/download
Supporting Information
SUPPORTING INFORMATION.docx

Gender Differences in the Italian Academic Landscape: Examining Inequalities within the Medical Area in the Last Decade

Magnano San Lio Roberta [1], Morini Mara [2], Di Rosa Enrico [2,3], Sinopoli Alessandra [2,3], Casigliani Virginia [2,4], Martella Manuela [2,5], Maugeri Andrea [1], Barchitta Martina [1], Agodi Maria Carmela [6], Picardi Ilenia [6], Agodi Antonella [1, 2*]

[1] Department of Medical and Surgical Sciences and Advanced Technologies "GF Ingrassia", University of Catania, 95123 Catania, Italy

[2] Italian Society of Hygiene, Preventive Medicine, and Public Health – SItI - Gender Prevention Working Group

[3] Department of Prevention, Local Health Authority Roma 1, 00193 Rome, Italy

[4] Department of Translational Research and of New Surgical and Medical Technologies, University of Pisa, Pisa, Italy

[5] Department of Public Health Science and Pediatrics, University of Turin, 10126 Torino, Italy

[6] Department of Political Science, University of Naples Federico II, Naples, Italy

on behalf of the Gender Prevention Working Group, SItI

Author's note

*Corresponding author: Antonella Agodi antonella.agodi@unict.it

Abstract

Gender equality remains a key focus in research and innovation policies. However, the academic sector still exhibits significant under-representation of women in leadership roles and horizontal segregation affecting career trajectories. This study examines gender inequalities in top positions and career progression within Italy's Medical Sciences sector (Area 06) from 2014 to 2023, through data from the Cineca portal, including details on academic roles, gender, region, and scientific-disciplinary sectors (SSDs). Gender disparity was evaluated using the Glass Ceiling Index (GCI) and the Glass Door Index (GDI). Data analysis up to 2023 shows a gender inversion in career progression: women dominate early career stages – accounted for 71.5% of research fellows and 57.1% of RTDA positions –, but men prevail in senior roles, with 61.8% associate professors and 71.8% full professors. This trend is consistent across most SSDs. Although temporal analysis indicates a reduction in gender disparities in top positions – with a GCI ranging from 2.3 in 2015 to 1.7 in 2023 - GCI values above one across all regions suggest persistent gender inequality. The GDI values, ranging from 1.3 in 2015 to 1.6 in 2023, exhibit considerable variability; however, overall, the disparities have worsened by 2023. These findings underscore the need for concrete measures to enhance equity and inclusivity in academia. Developing and implementing initiatives, programs, and policies that guarantee equal opportunities and resources for all researchers, irrespective of gender, is essential.

1. Introduction

Gender inequality remains a persistent issue in various professional fields, and academia is no exception [1, 2]. Despite regulatory interventions and gender mainstreaming policies, gender segregation remains persists, leading to the continued underrepresentation of women in top positions [3]. The European context provides a broader framework for analysing these issues, as gender disparity in academia is a well-documented phenomenon across the continent. ~~Despite various initiatives and policies aimed at promoting gender equality, many European countries still exhibit substantial gender gaps in higher education and research.~~ According to the European Commission's "She Figures 2021" report, women remain underrepresented in scientific and technical fields, and their progression to senior academic roles is significantly hindered compared to their male counterparts [4]. ~~Specifically, women face significant barriers in achieving gender parity in research and innovation. Although women now represent 48% of doctoral graduates in Europe, their representation decreases significantly in advanced research roles and leadership positions. Moreover,~~ Disparities are particularly evident in STEM (science, technology, engineering, and mathematics) fields, where women remain a minority in both education and employment. ~~For instance, in 2018, women accounted for only 32% of researchers in natural sciences and engineering.~~ Financial disparities also persist, as women researchers are less likely to receive competitive grants or lead major projects. ~~According to the *She Figures 2021* report, women comprised only 24% of applicants for European Research Council grants between 2014 and 2020, with lower success rates compared to men in several countries.~~ However, the composition of teams - particularly in terms of gender balance - can significantly enhance collective performance. Evidence suggests that gender diversity strengthens the effectiveness of workgroups within STEM fields. Therefore, it is essential not only to promote greater access for women to education and careers in STEM, but also to encourage a transformation in social and professional dynamics. This shift should aim to recognize gender diversity as a key driver of innovation, creativity, and sustainable growth across these sectors [5].

The Gender Equality Strategy 2020-2025 - adopted by the European Commission in March 2020 to set out the priorities and actions the EU intends to undertake during the 2020–2025 period to reduce gender inequalities - aims to create an ecosystem where women and men have equal opportunities to thrive in all aspects of academic and scientific life, contributing to a more inclusive and innovative Europe [6]. The gender gaps – especially in academia and research – reflect structural and cultural barriers, including unconscious biases, work-life balance challenges, and limited access to mentorship and networking opportunities. Addressing these disparities requires comprehensive approaches, such as targeted funding for women-led research, policies supporting work-life balance, and initiatives fostering inclusivity in traditionally male-dominated disciplines. In practice, these measures have already been incorporated into the Horizon Europe programme, which not only dedicates specific calls for proposals aimed at promoting gender equality in research teams, but also requires participating institutions to have a Gender Equality Plan (GEP) in place as an eligibility criterion for funding. Furthermore, efforts are being made to enhance the visibility of female role models in academia, which can provide inspiration and guidance to younger generations of women scientists [7].

In Italy, these disparities are evident in numerous aspects of academic life, including hiring practices, career progression, and access to research funding [8]. Recent research and reports indicate that, while women constitute a significant proportion of university graduates in Italy, their presence still dwindles at higher academic ranks [9]. ~~This trend mirrors the broader European pattern where, despite increasing numbers of female PhD graduates, the proportion of women in full professorship positions remains disproportionately low. Thus, gender inequalities manifest in the challenges women face in attaining top management and leadership positions in scientific institutions.~~ These inequalities also include gender-based differentiation throughout academic career paths in the current Italian university system, beginning from the recruitment stages [10-13]. Recent studies have revealed that, despite women often having more distinguished graduate and post-graduate careers and earning degrees faster than men, especially in STEM fields, they are predominantly found in temporary positions in

academia [14]. The percentage of female researchers hired on a permanent basis is significantly lower compared to their male counterparts, as it also happens at the European level.

~~The Gender Equality Strategy 2020-2025 by the European Commission highlights the need for continued action to bridge the gender gaps that persist across multiple sectors, particularly in academia and research. One of its key objectives is to promote gender balance in decision-making processes, ensuring that women have equal representation in leadership roles within academic and scientific institutions. This includes initiatives to increase female participation in research leadership and decision-making bodies, as well as supporting women's access to funding, mentorship, and networks that have traditionally been dominated by men. Additionally, the strategy emphasizes the importance of ensuring equal economic independence for both women and men. This includes measures to tackle the gender pay gap, improve access to funding for women researchers, and provide support for women to advance in their careers without being hindered by unequal access to opportunities. The strategy also stresses the need for policies that help combat unconscious biases and structural barriers that hinder women's academic progression. These barriers include work-life balance challenges, the lack of flexible working hours, and ingrained cultural norms that perpetuate gender stereotypes. The strategy also calls for gender-responsive research and policies, which would involve integrating gender perspectives into all stages of research and policymaking. This would ensure that gender equality is not only a theoretical goal but is embedded into practice, helping to address the systemic inequalities that limit women's success in scientific and academic careers.~~

In Italy, academic careers in research and teaching are structured into several roles: Full Professor, Associate Professor, Researcher (a position being phased out), Fixed-term Researcher Type B (RTDB) as per Art. 24, para. 3, letter b of Law 240/2010, Fixed-term Researcher Type A (RTDA) as per Art. 24, para. 3, letter a of Law 240/2010, and Research Fellow.

RTDB positions involve a non-renewable three-year contract, but researchers can transition directly to Associate Professor if they possess the National Scientific Qualification and receive a positive evaluation from the university (it is a tenure track position). RTDA positions have contract lasting

three years, renewable for an additional two years. Research fellows have contracts ranging from a minimum of one year to a maximum of three years, with a cumulative limit of six years for an individual in this role. As per the MIUR decree *"Integrazione del decreto del Ministro dell'istruzione, dell'università e della ricerca 1 settembre 2016, n. 662, recante la tabella di corrispondenza tra posizioni accademiche italiane ed estere ai sensi dell'articolo 18, comma 1, lettera b), della Legge 30 dicembre 2010, n. 240."*, which specifies the correspondence between Italian and foreign academic positions, ~~the alignment of Italian and foreign academic positions stipulates that~~ full professors correspond to GRADE A, associate professors to GRADE B, RTDB to GRADE C, and RTDA to GRADE D [15], as these GRADES are defined in the SHE Figures Report classification [4].

In addition to the roles mentioned, the position of Temporary Extraordinary Professor (as per Art. 1, para. 12 of Law 230/2005) remains in effect. This role involves a three-year contract that can be renewed for an additional three years and is funded by external entities for research activities. It is reserved for those who have obtained eligibility for the full professorship position or for individuals with high scientific and professional qualifications. Holders of these positions are entitled, for the duration of the contract, to the legal and economic treatment of full professors, with possible additional economic supplements, if provided for by the agreement [16].

The academic landscape in Italy has faced growing scrutiny over gender disparities in recent years, particularly within the medical field (Area 06 – Medicine). This study seeks to comprehensively analyse gender inequalities in academic medicine over the past decade, focusing on the period following the Gelmini Reform (Law 240/2010). This reform fundamentally reshaped the Italian academic system by introducing two fixed-term positions—the fixed-term contract A (RTDA) and the tenure-track fixed-term contract B (RTDB)—while abolishing the previously permanent role of researcher.

The primary aim of this study is to examine how these structural changes have influenced gender representation across different academic roles within medicine. It also aims to identify disparities across scientific-disciplinary sectors (Settori Scientifico-Disciplinari, SSD) and geographic regions

in Italy, shedding light on regional and sector-specific dynamics that impact gender equity in academic career progression. By doing so, the study provides a nuanced understanding of the systemic challenges and opportunities for promoting gender parity in Italian academic medicine.

2. Materials and methods

The study analysed comprehensive data on academic roles spanning the years 2014 to 2023, sourced from the Cineca portal (https://cercauniversita.mur.gov.it/php5/docenti/cerca.php?SESSION). This dataset included detailed records on academic positions, encompassing research fellows, fixed-term researchers (RTDA, RTDB), tenured researchers, associate professors, full professors, and temporary extraordinary professors. Each record provided information on academic roles, gender, affiliated university, region, and scientific-disciplinary sectors (SSD). Notably, data for research fellows were limited to the year 2023, while data for other positions spanned the entire period under study.

Certain categories of researchers were excluded from this analysis to ensure consistency and clarity. Specifically, data on full-time fixed-term researchers introduced under Law 79/2022, which replaced research fellowships in 2022, as well as researchers employed under Art. 1, para. 14, Law 230/05 prior to the differentiation into RTDA and RTDB categories, and data on non-tenured researchers, were omitted. This exclusion was necessary to maintain a uniform approach to analysing academic roles and to focus on positions with clear and consistent categorizations over the period.

For each academic role, data were initially examined by calculating gender proportions and the female-to-male ratio, providing insights into the distribution of men and women across various positions. To assess gender disparities, particularly in senior academic positions and recruitment pathways, the Glass Ceiling Index (GCI) was utilized. The GCI serves as a relative measure, comparing the proportion of women in academia at all levels (grades A, B, C, and D) to their representation in top academic positions (grade A) for a given year.

The GCI is computed using key gender-disaggregated variables for a given year Y, as follows:

- F_{AY} : Number of women full professors.
- M_{AY}: Number of men full professors.
- F_{BY}: Number of women associate professors.
- M_{BY}: Number of men associate professors.
- F_{CY} and F_{DY}: Number of women researchers (RTDB and RTDA, respectively).
- M_{CY} and M_{DY}: Number of men researchers (RTDB and RTDA, respectively).

These variables allow for a detailed examination of gender distribution across the academic hierarchy, from researchers to senior professors. The GCI quantitatively evaluates the extent to which women are underrepresented in higher academic positions relative to their overall presence in academia. According to the She Figures Report, the GCI is defined as follows [17]:

$$GCI = (F_{AY} + F_{BY} + F_{CY} + F_{DY}) / (F_{AY} + F_{BY} + F_{CY} + F_{DY} + M_{AY} + M_{BY} + M_{CY} + M_{DY}) \, / \, F_{AY} / (F_{AY} + M_{AY})$$

Where the numerator calculates the proportion of women among the total academic workforce (all grades combined: A, B, C, and D), accounting for both female (F_{AY}, F_{BY}, $F_{CY,}$ and F_{DY}) and male (M_{AY}, M_{BY}, $M_{CY,}$ and M_{DY}) staff. The denominator calculates the proportion of women at the grade A level (F_{AY}) relative to the total number of grade A professors (F_{AY} and M_{AY}). Accordingly, the GCI is a continuous metric ranging from 0 to infinity, providing insights into gender disparities in achieving the highest academic ranks. A GCI of 1 indicates gender parity in promotion opportunities. Women and men have equal chances of being promoted to grade A (Full Professor), reflecting a balanced academic system. A GCI below 1 suggests that women are overrepresented at grade A relative to their overall presence in academia. This scenario is rare and might occur in specific disciplines or contexts where targeted policies or historical trends have favoured women's advancement. A GCI above 1 reveals a glass ceiling effect, where women are underrepresented in grade A positions compared to their overall representation in academia. The higher the GCI value, the more significant the barrier for women in advancing to Full Professor roles.

The more innovative part of the analysis is the one quantitatively analysing gender disparities in the recruitment phase, where the effects of the introduction of the fixed term positions RTDA and RTDB deploys its effects. To this aim, we employed the GDI, an index recently introduced to reveal and measure the gender bias in academic recruitment [18]. It is calculated as the ratio between two percentages: the percentage of women in temporary research positions ($PW_{\leq D}$) and the percentage of women in positions that lead to stable academic careers (PW_{D}). This index provides insight into the progression of women from temporary roles to more permanent, stable academic positions. Specifically:

$$GDI = PW_{\leq D,Y} / PW_{DY} = F_{\leq D,Y} / F_{\leq D,Y} + M_{\leq D,Y} \Big/ F_{DY} / F_{DY} + M_{DY}$$

where F_{DY} (M_{DY}) denotes the number of women (men) in the position leading to stable academic roles in year Y, and $F_{\leq D,Y}$ ($M_{\leq D,Y}$) represents the number of women (men) in temporary research positions and the position leading to academic roles in year Y. Specifically, we computed the GDI value using the RTDB position as the initial stable academic role. Briefly, based on the relative presence of women in temporary research roles and positions leading to academic roles, the GDI measures the fraction that has attained stabilization. Like the GCI, the GDI ranges from 0 to infinity. A GDI equal to or less than 1 indicates that the percentage of women in the tenure track position (RTDB) is greater or equal compared to their percentage in temporary positions (RTDA). Conversely, a GDI above 1 signals the presence of a glass door that restricts women's entry, reflecting a recruitment process towards permanent positions in academia biased against women. The higher the GDI value, the stronger the impact of this glass door on entry into academia. It is important to note that in certain cases, the calculation of the GCI and GDI was not feasible due to the absence of key academic roles in the available data. Specifically, when one or more relevant positions were entirely missing, the indices could not be computed reliably. For instance, the absence of full professors made it impossible to calculate the GCI, which relies on comparing the representation of women at the

highest academic rank to their overall presence. Similarly, the GDI could not be determined in cases where either RTDA or RTDB positions were not present, as both are essential to assess transitions from temporary to permanent academic roles.

In 2023, our analysis employed a variety of methodological approaches to comprehensively investigate gender representation across academic levels. Descriptive statistics were used to calculate the percentages of males and females at each academic rank, alongside the computation of the GDI and the Glass Ceiling Index GCI to quantitatively measure disparities in recruitment and progression. To visualize these disparities, we utilized scissor diagrams to depict gender distributions across academic levels, illustrating the divergence in career trajectories between men and women. Geographic variations in gender disparities were analysed through choropleth maps, which displayed the GDI and GCI indices across Italy, providing a spatial representation of regional differences (i.e., the region Valle d'Aosta is not present because it does not host any university). The temporal dimension of gender disparities was explored using a longitudinal analysis covering the period from 2014 to 2023. This analysis employed line graphs to examine changes in GDI, GCI, and the female-to-male ratio over time, allowing for the identification of trends and shifts across the decade. Heatmaps were further applied to capture the evolution of GDI and GCI across scientific-disciplinary sectors (SSDs) and Italian regions, enabling a granular understanding of gender disparities within specific fields and locations. The SSD classification used in this analysis was aligned with the official categorizations provided by the Italian National University Council (CUN; https://www.cun.it/uploads/storico/settori_scientifico_disciplinari_english.pdf), as detailed in Table S1 ~~(Table 1).~~ Together, these methodological tools provided a robust framework for analysing and visualizing the complex dynamics of gender representation and disparities in academia.

3. Results

3.1. Gender disparities in Italian academic Medical Area in 2023

In 2023, women accounted for a total of 4,783 individuals across all academic roles, while men numbered 6,353, highlighting a marked gender imbalance. This imbalance is particularly evident when analyzing the distribution across different career stages. Women were more represented in early-career positions, such as research fellows (1,157 women vs. 462 men) and RTDA positions (751 women vs. 565 men). However, as academic rank increases, the trend reverses: men outnumber women in RTDB positions (625 men vs. 431 women), among researchers (557 men vs. 491 women), associate professors (2,371 men vs. 1,465 women), and even more so among full professors (1,722 men vs. 483 women). The disparity reaches its peak among temporary extraordinary professors, with 51 men compared to just 5 women.

This pattern is further illustrated by the "career scissor" diagram, a widely used representation of vertical segregation in academia. The diagram clearly shows the predominance of women in the initial stages of the academic path - 71.5% of research fellows and 57.1% of RTDA positions are held by women. However, the proportion of women steadily declines at each successive stage: 40.8% in RTDB roles, 46.8% among researchers, 38.2% among associate professors, and just 21.9% at the full professor level, compared to 78.1% men ~~The career scissor diagram – one of the most common representations of the phenomenon of vertical segregation in academia – confirms and depicts better this pattern, showing the predominance of women among research fellows (71.5% women vs. 28.5% men) and RTDA (57.1% women vs. 42.9% men). However, the curves showed how the proportion of women gradually decreased in the subsequent career phases, for RTDB (40.8% vs. 59.2), researchers (46.8% vs. 59.2%), associate professors (38.2% vs. 61.8%), and full professors (21.9% vs. 78.1%)~~ (**Figure 1**).

Figure 1. Career scissor diagram in Italian Medical Area in 2023

Interestingly, the gender distribution of Temporary Extraordinary Professors showed a predominance of males The greatest disparity is observed among temporary extraordinary professors, with 91.1% men and only 8.9% women (**Figure 2**).

Figure 2. Gender distribution of Temporary Extraordinary Professor in Italian Medical Area in 2023

Gender disparity is prevalent across most SSDs, with absolute numbers reported in Table S2. Specifically, a gender segregation with a clear predominance of men was observed for the following SSDs: MED/10 - Respiratory diseases, MED/11 – Cardiovascular diseases, MED/13 - Endocrinology, MED/14 - Nephrology, MED/18 - General surgery, MED/19 – Plastic surgery, MED/20 – Paediatric surgery, MED/21 – Thoracic surgery, MED/22 – Vascular surgery, MED/23 – Heart surgery, MED/24 - Urology, MED/27 - Neurosurgery, MED/28 - Oral diseases and dentistry, MED/29 - Maxillofacial surgery, MED/30 - Eye diseases, MED/31 - Otorhinolaryngology, MED/33 - Musculoskeletal system diseases, MED/37 - Neuroradiology, MED/40 - Obstetrics and gynaecology, MED/41 - Anaesthesiology. Conversely, there were other fields where gender segregation is less pronounced, and women equal or surpass the percentage of men (e.g., MED/01 - Medical statistics, MED/02 - Medical history, MED/03 - Medical genetics, MED/04 - Experimental medicine and pathophysiology, MED/05 - Clinical pathology, MED/07 - Microbiology and clinical microbiology, MED/38 - General and subspecialty paediatrics, MED/39 - Child neuropsychiatry, MED/42 - Hygiene and public health, MED/45 - Nursing sciences: general, clinical and paediatric, MED/47 - Medical and biotechnology laboratory techniques, MED/48 - Midwifery, MED/49 - Neuropsychiatric and rehabilitation nursing sciences, MED/50 - Food sciences and dietetics). However, for the SSDs MED/38, MED/39, MED/42, MED/45, MED/49, and MED/50, there continues to be a predominance of men in the role of full professor. Moreover, in general, women

were more frequently represented in Research Fellow and RTDA roles but were underrepresented at higher academic levels. However, this pattern does not hold in SSDs MED/22, MED/24, MED/27, MED/33, and MED/37, where men outnumber women in all academic positions. Notably, in SSD MED/20, men held a larger proportion of Research Fellow positions, while women were predominantly found in RTDA roles. Further details are shown in **Table** 1 ~~2~~.

Table 1 ~~2~~.. Gender disparity within SSDs

SSD	Gender	Research Fellows	RTDA	RTDB	Tenured Researches	Associate Professors	Full Professors	Temporary Extraordinary Professor
MED/01	Female	73.3%	68.8%	50.0%	50.0%	63.8%	51.2%	
	Male	26.7%	31.3%	50.0%	50.0%	36.2%	48.8%	
MED/02	Female	80.0%	66.7%	50.0%	40.0%	46.7%	66.7%	
	Male	20.0%	33.3%	50.0%	60.0%	53.3%	33.3%	
MED/03	Female	83.9%	64.7%	68.4%	82.4%	58.2%	39.6%	
	Male	16.1%	35.3%	31.6%	17.6%	41.8%	60.4%	
MED/04	Female	76.0%	74.7%	63.9%	72.9%	61.9%	40.8%	0.0%
	Male	24.0%	25.3%	36.1%	27.1%	38.1%	59.2%	100.0%
MED/05	Female	85.0%	88.9%	72.7%	73.3%	70.5%	46.7%	
	Male	15.0%	11.1%	27.3%	26.7%	29.5%	53.3%	
MED/06	Female	76.7%	50.0%	40.0%	50.0%	30.6%	15.0%	33.3%
	Male	23.3%	50.0%	60.0%	50.0%	69.4%	85.0%	66.7%
MED/07	Female	69.4%	76.3%	55.6%	63.6%	71.6%	50.0%	
	Male	30.6%	23.7%	44.4%	36.4%	28.4%	50.0%	
MED/08	Female	63.3%	50.0%	52.4%	57.7%	44.9%	32.0%	
	Male	36.7%	50.0%	47.6%	42.3%	55.1%	68.0%	
MED/09	Female	68.4%	48.3%	36.2%	51.4%	33.9%	16.9%	0.0%
	Male	31.6%	51.7%	63.8%	48.6%	66.1%	83.1%	100.0%
MED/10	Female	81.0%	64.3%	30.0%	37.5%	39.5%	19.4%	0.0%
	Male	19.0%	35.7%	70.0%	62.5%	60.5%	80.6%	100.0%
MED/11	Female	76.3%	54.8%	19.4%	35.5%	29.7%	8.2%	0.0%
	Male	23.7%	45.2%	80.6%	64.5%	70.3%	91.8%	100.0%
MED/12	Female	76.7%	45.5%	50.0%	71.4%	26.6%	19.0%	0.0%
	Male	23.3%	54.5%	50.0%	28.6%	73.4%	81.0%	100.0%
MED/13	Female	87.1%	60.0%	63.6%	35.7%	48.5%	19.0%	
	Male	12.9%	40.0%	36.4%	64.3%	51.5%	81.0%	
MED/14	Female	83.3%	47.1%	50.0%	50.0%	27.3%	14.3%	50.0%
	Male	16.7%	52.9%	50.0%	50.0%	72.7%	85.7%	50.0%
MED/15	Female	67.6%	69.0%	36.4%	52.6%	41.0%	12.5%	0.0%
	Male	32.4%	31.0%	63.6%	47.4%	59.0%	87.5%	100.0%
MED/16	Female	80.0%	61.5%	30.0%	66.7%	54.4%	19.2%	
	Male	20.0%	38.5%	70.0%	33.3%	45.6%	80.8%	
MED/17	Female	77.8%	50.0%	38.9%	56.0%	37.9%	25.0%	0.0%
	Male	22.2%	50.0%	61.1%	44.0%	62.1%	75.0%	100.0%
MED/18	Female	76.2%	30.4%	23.9%	23.1%	11.3%	3.5%	0.0%
	Male	23.8%	69.6%	76.1%	76.9%	88.7%	96.5%	100.0%
MED/19	Female	66.7%	40.0%	26.7%	20.0%	12.5%	3.8%	0.0%
	Male	33.3%	60.0%	73.3%	80.0%	87.5%	96.2%	100.0%
MED/20	Female	33.3%	66.7%	33.3%	40.0%	9.5%	14.3%	
	Male	66.7%	33.3%	66.7%	60.0%	90.5%	85.7%	
MED/21	Female	100.0%	50.0%	50.0%	0%	7.7%	5.3%	
	Male	0%	50.0%	50.0%	100.0%	92.3%	94.7%	
MED/22	Female	0%	44.4%	0%	18.2%	12.2%	0%	
	Male	100.0%	55.6%	100.0%	81.8%	87.8%	100.0%	
MED/23	Female	66.7%	57.1%	0%	0%	2.4%	3.6%	
	Male	33.3%	42.9%	100.0%	100.0%	97.6%	96.4%	
MED/24	Female	40.0%	7.1%	6.7%	0%	2.1%	2.2%	
	Male	60.0%	92.9%	93.3%	100.0%	97.9%	97.8%	
MED/25	Female	60.6%	40.9%	38.5%	37.5%	43.1%	24.4%	0.0%
	Male	39.4%	59.1%	61.5%	62.5%	56.9%	75.6%	100.0%
MED/26	Female	64.0%	47.0%	39.5%	50.0%	41.1%	17.4%	100.0%
	Male	36.0%	53.0%	60.5%	50.0%	58.9%	82.6%	0.0%
MED/27	Female	37.5%	20.0%	12.5%	16.7%	5.1%	3.1%	0.0%
	Male	62.5%	80.0%	87.5%	83.3%	94.9%	96.9%	100.0%
MED/28	Female	60.4%	31.3%	31.7%	36.8%	22.2%	21.5%	0.0%
	Male	39.6%	68.8%	68.3%	63.2%	77.8%	78.5%	100.0%
MED/29	Female	50.0%	14.3%	22.2%	18.2%	14.3%	0%	0.0%
	Male	50.0%	85.7%	77.8%	81.8%	85.7%	100.0%	100.0%

SSD	Gender	Research Fellows	RTDA	RTDB	Tenured Researches	Associate Professors	Full Professors	Temporary Extraordinary Professor
MED/30	Female	56.0%	41.4%	53.8%	39.0%	24.7%	4.7%	0.0%
	Male	44.0%	58.6%	46.2%	61.0%	75.3%	95.3%	100.0%
MED/31	Female	90.9%	22.2%	17.6%	23.5%	13.6%	0%	0.0%
	Male	9.1%	77.8%	82.4%	76.5%	86.4%	100.0%	100.0%
MED/32	Female	75.0%	16.7%	100.0%	62.5%	36.7%	37.5%	
	Male	25.0%	83.3%	0%	37.5%	63.3%	62.5%	
MED/33	Female	33.3%	22.2%	3.2%	11.1%	9.3%	0%	0.0%
	Male	66.7%	77.8%	96.8%	88.9%	90.7%	100.0%	100.0%
MED/34	Female	43.8%	54.5%	40.0%	57.1%	35.1%	25.9%	
	Male	56.3%	45.5%	60.0%	42.9%	64.9%	74.1%	
MED/35	Female	65.6%	62.5%	68.2%	64.3%	46.6%	32.3%	0.0%
	Male	34.4%	37.5%	31.8%	35.7%	53.4%	67.7%	100.0%
MED/36	Female	62.2%	35.9%	24.4%	50.0%	35.7%	16.3%	100.0%
	Male	37.8%	64.1%	75.6%	50.0%	64.3%	83.7%	0.0%
MED/37	Female	0%	20.0%	10.0%	0%	31.6%	21.1%	0.0%
	Male	100.0%	80.0%	90.0%	100.0%	68.4%	78.9%	100.0%
MED/38	Female	76.4%	73.8%	51.4%	58.8%	51.6%	23.3%	50.0%
	Male	23.6%	26.2%	48.6%	41.2%	48.4%	76.7%	50.0%
MED/39	Female	78.9%	91.7%	68.8%	72.7%	64.9%	30.0%	
	Male	21.1%	8.3%	31.3%	27.3%	35.1%	70.0%	
MED/40	Female	88.9%	46.2%	44.8%	63.4%	38.1%	13.1%	0.0%
	Male	11.1%	53.8%	55.2%	36.6%	61.9%	86.9%	100.0%
MED/41	Female	53.3%	41.7%	30.0%	45.9%	23.9%	12.5%	0.0%
	Male	46.7%	58.3%	70.0%	54.1%	76.1%	87.5%	100.0%
MED/42	Female	75.0%	63.0%	53.6%	65.0%	63.2%	36.8%	0.0%
	Male	25.0%	37.0%	46.4%	35.0%	36.8%	63.2%	100.0%
MED/43	Female	76.5%	35.3%	36.8%	40.0%	37.8%	23.3%	0.0%
	Male	23.5%	64.7%	63.2%	60.0%	62.2%	76.7%	100.0%
MED/44	Female	52.0%	53.3%	35.7%	60.0%	44.4%	25.0%	0.0%
	Male	48.0%	46.7%	64.3%	40.0%	55.6%	75.0%	100.0%
MED/45	Female	88.9%	57.9%	28.6%		56.7%	75.0%	
	Male	11.1%	42.1%	71.4%		43.3%	25.0%	
MED/46	Female	76.4%	87.7%	64.3%	70.0%	70.1%	52.8%	
	Male	23.6%	12.3%	35.7%	30.0%	29.9%	47.2%	
MED/47	Female		100.0%		100.0%	100.0%		
	Male		0.0%		0.0%	0.0%		
MED/48	Female	55.6%	70.0%	10.0%	33.3%	73.3%	57.1%	
	Male	44.4%	30.0%	90.0%	66.7%	26.7%	42.9%	
MED/49	Female	77.1%	73.7%	77.3%	45.5%	52.5%	34.6%	
	Male	22.9%	26.3%	22.7%	54.5%	47.5%	65.4%	
MED/50	Female	64.1%	79.2%	53.6%	45.5%	45.5%	28.9%	0.0%
	Male	35.9%	20.8%	46.4%	54.5%	54.5%	71.1%	100.0%

3.2.Gender disparities across Italian regions in 2023

We also analysed gender disparity across Italian regions using the GCI and GDI, while absolute figures for each academic position are provided in Table S3. Our findings indicate a presence of a glass ceiling effect in all Italian regions, suggesting that women are underrepresented in Full Professor positions compared to other academic roles. Excluding regions for which the available data did not fulfil the conditions necessary for the calculation of the GCI (i.e., Basilicata, Molise, and Valle d'Aosta), Sardinia, Liguria, and Veneto displayed the highest levels of gender inequality,

disadvantaging women, with GCI values of 2.9, 2.5, and 2.3, respectively. Conversely, the lowest GCI values were observed in Piedmont (1.4), Trentino-Alto Adige (1.3), and Friuli-Venezia Giulia (1.2) (**Figure 3**).

Figure 3. Gender disparity in Italian Medical Area in 2023 according to Glass Ceiling Index

According to the GDI, only the regions of Molise and Trentino-Alto Adige reported a value below one, highlighting that in the tenure track position, the percentage of women is higher (or equal) compared to the percentage of women in temporary positions. This result must be considered cautiously as it could be only due to the low number of professionals in the academic roles in these regions. By contrast, the highest GDI values were detected in Abruzzo (2.7), Apulia (2.2), Sardinia (2.1) (**Figure 4**).

Figure 4. Gender disparity in Italian Medical Area in 2023 according to Glass Door Index

3.3. Gender disparities in Italian academic Medical Area from 2014 to 2023

We next assessed the temporal trend of female-to-male ratio from 2015 to 2023, according to academic roles (**Figure 5**). Over time, there has been a gradual rise in female-to-male ratios, suggesting an increasing presence of women across all academic positions. This increase has been moderate across most roles, apart from RTDA positions, where a more significant rise has been observed. It is worthwhile to note that gender parity (a female-to-male ratio of 1) has not been achieved, except in the RTDA category.

Figure 5. Temporal trend of female-to-male ratio from 2015 to 2023

We next assessed the temporal trend - from 2015 to 2023 – of gender disparities in Italian academic Medical Area. According to GCI, the temporal analysis showed a reduction in female disparities in academic top positions, from 2.3 in 2015 to 1.7 in 2023. Moreover, GDI increased from 1.3 in 2015 to 1.6 in 2023 (**Figure 6**).

Figure 6. Temporal trends of gender disparities from 2015 to 2023 according to GCI and GDI

We also examined temporal trends in gender disparities within regions using GDI and GCI. In general, the GCI remained above 1 across all regions and throughout the entire period under analysis, with the exception of Molise and Trentino-Alto Adige in 2017, which recorded values of 0.3 and 0.8, respectively. However, it is important to highlight that, for these regions - along with Basilicata - the available data did not consistently meet the criteria required for GCI calculation over the full time span considered. Among the remaining regions, median GCI values ranged from 1.6 in Piedmont to 2.9 in Sardinia, while a general tendency toward decreasing vertical segregation emerged over the analysed period. Specifically, Friuli-Venezia Giulia demonstrated a marked improvement, with the GCI decreasing from 4.0 in 2015 to 1.2 in 2023. In contrast, Sardinia represented the only exception, showing a worsening trend in vertical segregation, with the GCI rising from 1.7 in 2015 to 2.9 in 2023.

The GDI values, by contrast, showed heterogeneous trends between 2015 and 2023, with most regions displaying an increased level of disparity disadvantaging women by 2023. Caution is advised when interpreting the indices for regions with a limited number of professionals across academic roles - such as Molise and Trentino-Alto Adige - as small sample sizes may affect the robustness of the estimates. Among the regions with complete data over the entire analysis period, median GDI values ranged from 0.8 in Marche to 2.4 in Friuli-Venezia Giulia. Further details can be found in Figures S1 and S2. ~~**Figure 7** and **Figure 8**.~~

~~**Figure 7. Temporal trends of gender disparities within Regions according to GCI**~~

~~**Figure 8. Temporal trends of gender disparities within Regions according to GDI**~~

Next, we assessed temporal trends of gender disparities within SSDs according to GDI and GCI. In general, the GCI usually reported a value greater than one, with some exceptions: SSD MED/02 from 2021 to 2023, MED/21 from 2014 to 2016, MED/23 in 2016 and from 2018 to 2020, MED/45 from 2014 to 2023, MED/47 in 2017, and MED/48 in 2014-2015. Among the SSDs with complete data, the highest median GCI value was observed in MED/30 (6.2), while the lowest was recorded in MED/45. For a detailed overview of the trend in each SSD, please refer to Figure S3. When considering the GDI, the situation appeared more heterogeneous, warranting an SSD-by-SSD analysis. Among the SSDs with complete data, the highest median GDI value was found in MED/11 (1.9), while the lowest was observed in MED/35 (0.9). Notably, MED/45 - already characterized by a relatively high proportion of women across academic roles (see paragraph 3.1) - reported a GCI of 0.7 in 2023, consistent with its 2014 value, and a GDI of 2.0, which represents a sharp increase from 0.3 in 2015. Further details are provided in Figure S4. ~~**Figure 9 and Figure 10.**~~

~~**Figure 9. Temporal trends of gender disparities within SSD according to GCI**~~

~~**Figure 10. Temporal trends of gender disparities within SSD according to GDI**~~

4. Discussion

Discrimination against women in the workplace remains a significant challenge, despite ongoing efforts to promote gender equality. According to the European Institute for Gender Equality (EIGE),

gender segregation and discrimination persist across various professional fields, with women facing systemic barriers to career advancement and leadership positions [1]. The "Gender Equality Index 2020" report by EIGE highlights that women are underrepresented in decision-making roles and experience a substantial pay gap compared to their male counterparts. This disparity is even more pronounced in sectors such as STEM, where women tend to be concentrated in lower-paying, junior roles, while senior leadership roles remain predominantly male [3]. Additionally, the European Commission's "She Figures 2021" report reveals that women are disproportionately affected by precarious employment conditions and have limited access to research funding and career development opportunities in academia [4]. Scientific evidence further underscores these disparities [19] highlighting the multifaceted nature of these disparities, which manifest in different forms across academic disciplines and career stages [20-24]. Research also indicates that gender disparities are not uniform across disciplines. However, even in more gender-balanced fields, women tend to face significant barriers when it comes to attaining senior academic positions and receiving recognition for their work [25]. Moreover, research by the European Commission demonstrates that unconscious biases and structural barriers, such as the lack of family-friendly policies and support for work-life balance, contribute to the ongoing discrimination against women in the workplace [6].

The use of the GCI and GDI has allowed us to assess gender disparities in the Italian academic medical field. Specifically, in the context of the Italian academic medical field, these indices serve as valuable tools for identifying barriers that women face in reaching leadership roles and their participation in high-ranking academic positions. The GCI measures the extent to which women face obstacles in advancing to top leadership positions, reflecting the metaphorical "ceiling" that limits their progression. In the context of Italian academia, studies have demonstrated that while women may achieve significant representation at lower academic levels, they tend to be underrepresented in senior roles such as professors, department heads, and deans. The GCI helps quantify this disparity, indicating how the progression of women in academia slows down as they move up the academic hierarchy. On the other hand, the GDI assesses the barriers women encounter when attempting to

enter academic fields or institutions, essentially evaluating how open or closed the "door" is to women at the onset of their academic careers. It looks at factors like recruitment practices, access to research funding, and the gendered dynamics of hiring decisions. In the Italian medical academic sector, evidence from the GDI shows that while women are well-represented in medical education, their participation drops in higher positions, which is influenced by unconscious bias, gendered perceptions of leadership, and institutional structures that favour men. In Italy, these indices have been instrumental in demonstrating that despite various initiatives for gender equality, there remain significant gender gaps in medical academia. The gender imbalances are not only seen in leadership roles but also in areas like research funding allocation and career advancement opportunities. By using the GCI and GDI, these studies highlight the need for policies that promote gender equality at all stages of an academic career—ensuring that women are not only able to enter the field but also have the support and opportunities to thrive and lead in their respective fields. Incorporating these tools into research also opens the door for more targeted interventions. For example, using the GCI and GDI can guide institutions to implement specific gender equality measures, such as mentorship programs, family-friendly policies, and transparent recruitment processes, that aim to dismantle the structural and cultural barriers that contribute to gender disparity in academic medicine. These indices provide a clear and quantifiable way to track progress and design more effective strategies to foster gender equity within the medical academic sector. The aim was to test the hypothesis that, also in this area, the gender gap in academic careers is not going to disappear in a relatively short time. As it has been shown by recent research findings the issue is not just that of waiting the time needed to see the new generations of women that have entered academia break the glass ceiling. The gender gap is self-sustaining through different mechanisms at different stages of the academic career, as the glass door effect revealed is happening at the transition from temporary positions to the tenure track position, after its recent introduction in the Italian university system [10, 18, 26, 27] by the latest general reform of the Italian university system (Law 240/2010, better known as the Gelmini Reform). Our findings confirm the hypothesis for the medical area, showing that a higher number of women in early career

stages does not correspond to a proportionate rate of women in tenure track positions. By contrast, as the percentage of women in the initial stages increases, the presence of obstacles to stabilization becomes more evident. Despite regulatory interventions and gender mainstreaming policies, the career scissor diagram for 2023 clearly illustrates the permanence of the decline in the representation of women as they advance through academic ranks, culminating in a significant underrepresentation at the full professor level. The temporal trend analysis from 2014 to 2023 offers a glimmer of hope, revealing a gradual increase in the female-to-male ratio across academic roles and a reduction in gender disparities in leadership positions. However, the pace of change remains slow, and achieving gender parity is still a distant goal for most academic roles. Furthermore, as Ferree and Purkayastha suggest, the decrease of a disadvantage in the transition from the intermediate (B) to the final level (A), is not unambiguously signalling greater gender equality to the extent that, on the one hand, women who are at the first level are still less likely to become tenured researchers than their male colleagues and, on the other hand, this decrease could be due precisely to the discrimination that women experience in the previous step [28]. Gender discrimination in the workplace often leads women to delay childbearing, fearing that employers may be less willing to hire, promote, or support working mothers. Many women also choose to leave their jobs after childbirth to care for their children, which further influences their decisions about starting a family. The widespread fear of discrimination against pregnant women and women with young children is closely linked to their choices to postpone or avoid having children [29].

Through the introduction of the GDI, the analysis empirically highlights the existence of gender differentiation processes in academic career paths within the medical area, starting from the recruitment phases. Specifically, the career scissor diagram varies significantly across different disciplines, and the comparison between GDI values before the reform and after the reform shows that, following the Gelmini reform, there has been a systematic relative decrease in the number of women accessing stable academic positions [18]. Comparison between SSDs has revealed disciplines

that historically show a predominance of the male gender, as especially surgical specialties. The higher percentage of men in these sectors may be attributed to various historical, cultural, and social factors. Traditionally, many of these fields have been perceived as more technical or less compatible with roles historically assigned to women. This may have led to lower initial participation of women and, consequently, to a persistence of gender disparity. Additionally, structural barriers and gender bias, the lack of female role models, and traditional gender expectations as well as the shortage in services regarding family responsibilities may have contributed to discouraging them from pursuing or continuing careers in these fields. Conversely, other disciplines, such as nursing sciences, show a predominance of women, although horizontal segregation persists.

The observed trend is consistent with broader European patterns, where gender disparities in academia are well-documented. ~~The observed predominance of women in early career stages, such as research fellows and RTDA, contrasts sharply with their dwindling presence in senior positions, underscoring the persistence of structural barriers and potentially unconscious biases that hinder women's career progression~~. As noted in several European studies, women are often underrepresented in higher-ranking positions, despite their significant contributions in early career stages [4]. For example, in the "She Figures 2021" report, women account for just 29% of full professors in Europe, despite representing nearly half of all doctoral graduates and postdoctoral researchers [4]. The predominance of women in the early career stages, particularly in research-focused roles, may be partly attributed to factors such as greater flexibility in these positions, which are sometimes more accessible for individuals balancing work and family responsibilities. However, as careers progress, the proportion of women drops substantially in response to institutional and cultural barriers. Women’s advancement is often slowed by the so-called "leaky pipeline," a phenomenon where women enter academia in equal or greater numbers than men but face increasing barriers that prevent them from reaching senior leadership roles. This includes not only biased career evaluation systems but also the challenges posed by work-life balance issues, insufficient mentorship, and unequal access

to professional networks, which are often critical for career advancement [7]. Geographical disparities further compound this issue, with regions like Sardinia, Liguria, and Veneto exhibiting the highest gender inequalities. In contrast, regions such as Piedmont and Trentino-Alto Adige show more equitable distributions, indicating regional variations in the effectiveness of gender equality policies. The GCI and GDI reinforce the notion that systemic barriers remain prevalent, with few regions demonstrating substantial progress in achieving gender parity in recruitment and promotion processes. The temporal trend analysis from 2014 to 2023 offers a glimmer of hope, revealing a gradual increase in the female-to-male ratio across academic roles and a reduction in gender disparities in leadership positions. However, the pace of change remains slow, and achieving gender parity is still a distant goal for most academic roles. As Ferree and Purkayastha suggest, the decrease of a disadvantage between adjacent levels, and more particularly in the transition from the intermediate (B) to the final level (A), does not necessarily mean greater gender equality to the extent that, on the one hand, women who are at the first level are still less likely to become tenured researchers than their male colleagues and, on the other hand, this decrease could be due precisely to the discrimination that women experience in the previous step [28]. The regional distribution of inequalities also suggests possible hypotheses about the gender distribution: women may be overrepresented in the highest positions in regions that are less desirable for logistic and prestige reasons as the case of Molise (a mountainous region with logistic difficulties, especially during the winter season) may suggest – although small numbers in the data call for cautious interpretations.

The study has also several limitations. First of all, we considered the lack of data on the number of research fellows in the years preceding 2023. As a result, this category has been excluded from the calculation of the GCI and GDI, potentially leading to an incomplete picture of gender inequality in early career stages. Additionally, the lack of data on the different academic positions did not allow for a complete calculation of the GCI and GDI indices, both at the regional level and across the SSDs. Moreover, the emphasis on quantitative data might overlook qualitative aspects of gender discrimination, such as personal experiences and organizational cultures that also play significant

roles in shaping women's career trajectories through context specific social mechanisms [30]. The study highlights that gender disparities are not uniform across disciplines. However, the research could benefit from a more detailed analysis of why certain fields are more gender-balanced than others, which might offer insights into more effective gender equality strategies. While the study mentions unconscious biases and structural barriers and suggests possible contextual mechanisms being at work in different regions, further research could delve deeper into these aspects to offer more concrete recommendations on how to address them [30].

5. Conclusion

In conclusion, while there have been notable advancements in gender equality in the Italian academic medical field, persistent inequalities continue to hinder the full integration and progression of women in the sector. These inequalities are evident not only in the underrepresentation of women in leadership roles, such as senior professors, heads of departments, and academic deans, but also in the unequal distribution of research funding and access to career advancement opportunities. Despite various initiatives aimed at promoting gender equality, these disparities remain entrenched due to both visible and invisible structural barriers that continue to favour male counterparts. Our findings suggest that while the glass ceiling remains a significant issue, there is also a pressing need to address the *glass door* phenomenon—the barriers preventing women from entering academic medicine in the first place. Factors such as unconscious bias in recruitment practices, the prioritization of male-dominated networks, and societal expectations surrounding gender roles contribute to this uneven playing field. These issues require a multi-faceted response that targets both the entry-level barriers and the challenges faced by women as they progress through their academic careers. More robust and targeted strategies are necessary to dismantle these structural barriers. Institutional policies should focus not only on ensuring fair and equitable recruitment practices but also on promoting transparent and inclusive promotion processes. This involves revising performance evaluation metrics that

traditionally favour male-dominated research areas and ensuring that caregiving responsibilities, which disproportionately fall on women, are not seen as obstacles to career progression. Mentorship programs, networking opportunities, and leadership training tailored specifically for women can also serve as crucial steps in addressing the systemic biases that persist in the field. Furthermore, fostering an inclusive academic environment requires cultural shifts within academic institutions. This includes promoting work-life balance policies, supporting parental leave, and creating family-friendly working conditions to reduce the gendered expectations placed on women. These cultural changes should be coupled with comprehensive policy implementation and sustained monitoring to track progress toward achieving gender equality. The road to achieving lasting gender equality in Italian academic medicine is undeniably challenging, as it requires overcoming deeply rooted biases, norms, and practices. However, it is a crucial goal that will not only benefit women but also enrich the academic medical community as a whole. Studies consistently show that diversity in leadership fosters better decision-making, more innovative research, and improved outcomes across various sectors, including healthcare. Therefore, addressing gender inequality is not just a matter of social justice but also an imperative for improving the quality of education and research in the medical field. Achieving true gender equality requires continuous, multi-dimensional efforts that include policy reforms, cultural transformation, and institutional support. The success of these efforts will depend on the willingness of institutions to recognize and address the systemic biases that perpetuate inequality, ultimately creating a more inclusive and equitable environment that allows all individuals, regardless of gender, to reach their full potential in academia.

Acknowledgments

The authors thank editor and our anonymous reviewers for their important feedback and guidance. The authors also thank the Gender Prevention Working Group of the Italian Society of Hygiene, Preventive Medicine, and Public Health - SItI.

Abbreviations

GCI Glass Ceiling Index

GDI Glass Door Index

RTDA Fixed-term Researcher Type A

RTDB Fixed-term Researcher Type B

SSD Scientific-disciplinary sector (Settore scientifico-disciplinare)

References

1. European Institute for Gender Equality, EIGE. Gender Equality in Academia and Research GEAR tool step. 2022. Available at: https://eige.europa.eu/sites/default/files/documents/20220795_pdf_mh0922276enn_002.pdfby-step guide.
2. Farina, S., Iafrate, R., and Boccia, S. 2023. "Unveiling the gender gap: exploring gender disparities in European academic landscape." Lancet Reg Health Eur 34:100752. doi: 10.1016/j.lanepe.2023.100752.
3. European Institute for Gender Equality, EIGE. Gender Equality Index 2020: Key Findings for the EU. Available at: https://eige.europa.eu/sites/default/files/documents/20203354_mhag20029enn_002.pdf
4. European Commission, 2021. She Figures 2021, Gender in research and innovation: statistics and indicators. Available at: https://op.europa.eu/it/publication-detail/-/publication/67d5a207-4da1-11ec-91ac-01aa75ed71a15.
5. Beroíza-Valenzuela F, Natalia S-G, and Huepe D. Bridging gaps: the role of gender and team composition in collective intelligence within STEM education. Cogent Education. 2025;12(1):2439655. doi: 10.1080/2331186X.2024.2439655.

6. European Commission, 2020. A Union of Equality: Gender Equality Strategy 2020-2025. Available at: https://eur-lex.europa.eu/legal-content/EN/TXT/PDF/?uri=CELEX:52020DC0152

7. Herschberg C., Benschop Y. e van den Brink M. 2018. Precarious postdocs: A comparative study on recruitment and selection of early-career researchers.

8. Liccardo A., Agodi MC., Picardi I, Pisanti O., Masullo MR and Gargano A. 2016. Primo bilancio di genere dell'Ateneo federiciano, Napoli, FedOAPress.

9. Celletti A., Costantini P, Primeri E, Romagnosi S. I focus del rapporto ANVUR 2023. Analisi di genere. 2023.

10. Murgia A, Poggio B. 2018. Gender and Precarious Research Careers: A Comparative Analysis. https://doi.org/10.4324/9781315201245

11. Picardi I., 2016. Per una dimensione di genere nella valutazione della ricerca, in Avveduto S. et al. (a cura di), Scienza, genere e società. 2016. Prospettive di genere in una società che si evolve, IRPPS Monografie.

12. Picardi I, Agodi M.C, 2017. Il mentoring come strumento di de-costruzione delle strutture di genere riprodotte nell'università e la ricerca, in Murgia A. e Poggio B. (a cura di), Prospettive interdisciplinari su formazione, università, lavoro. 2017.

13. Agodi, M.C, and Picardi I. 2016. "Il mentoring come progetto trasformativo della struttura di genere dell'accademia: il programma pilota di GENOVATE@UNINA."

14. Consorzio Interuniversitario Almalaurea. FOCUS GENDER GAP 2023. Available at: https://www.almalaurea.it/sites/default/files/2023-03/FOCUS-GENDER-GAP_2023.pdf

15. Ministero dell'Istruzione e del Merito. Integrazione del decreto del Ministro dell'istruzione, dell'università e della ricerca 1 settembre 2016, n. 662, recante la tabella di corrispondenza tra posizioni accademiche italiane ed estere ai sensi dell'articolo 18, comma 1, lettera b), della Legge 30 dicembre 2010, n. 240. Available at: https://www.miur.gov.it/-/integrazione-del-decreto-del-ministro-dell-istruzione-dell-universita-e-della-ricerca-1-settembre-2016-n-662-recante-la-tabella-di-corrispondenza-tra

16. Ministero dell’Istruzione e del Merito. Reclutamento delle Università. Available at: https://www.miur.gov.it/reclutamento-nelle-universita#:~:text=Ricercatori%20a%20tempo%20indeterminato%3A,3%20della%20Legge%20210%2F1998.

17. European Commission, 2015. She Figures 2015. Gender in research and innovation: statistics and indicators. Available at: https://op.europa.eu/it/publication-detail/-/publication/67d5a207-4da1-11ec-91ac-01aa75ed71a1

18. Picardi I. The Glass Door Index: revealing the gendered impact of the Gelmini reform on the access to the academic profession. 2019. Soc. Sci. 2019, 8, 160. https://doi.org/10.3390/socsci8050160

19. Moss-Racusin CA, Dovidio JF, Brescoll VL, Graham MJ, Handelsman J. Science faculty's subtle gender biases favor male students. Proc Natl Acad Sci U S A. 2012;109(41):16474-9. Epub 20120917. doi: 10.1073/pnas.1211286109. PubMed PMID: 22988126; PubMed Central PMCID: PMCPMC3478626.

20. Llorens, A., A. Tzovara, L. Bellier, I. Bhaya-Grossman, A. Bidet-Caulet, W. K. Chang, Z. R. Cross, R. Dominguez-Faus, A. Flinker, Y. Fonken, M. A. Gorenstein, C. Holdgraf, C. W. Hoy, M. V. Ivanova, R. T. Jimenez, S. Jun, J. W. Y. Kam, C. Kidd, E. Marcelle, D. Marciano, S. Martin, N. E. Gender bias in academia: A lifetime problem that needs solutions. Neuron, Volume 109, Issue 13, 2047 – 2074.

21. Lombardo E., Naldini M., Poggio B. Gender Inequalities in Academia: Multiple Approaches to Closing the Gap. 2024. https://doi.org/10.3280/sl2024-168001

22. Galvin DJ, Anderson SC, Marolf CJ, Schneider NG, Liebl AL. Comparative analysis of gender disparity in academic positions based on U.S. region and STEM discipline. PLoS One. 2024;19(3):e0298736. Epub 20240320. doi: 10.1371/journal.pone.0298736. PubMed PMID: 38507318; PubMed Central PMCID: PMCPMC10954166.

23. Bendels MHK, Müller R, Brueggmann D, Groneberg DA. Gender disparities in high-quality research revealed by Nature Index journals. PLoS One. 2018;13(1):e0189136. Epub 20180102. doi: 10.1371/journal.pone.0189136. PubMed PMID: 29293499; PubMed Central PMCID: PMCPMC5749692.

24. Ross, M. B., B. M. Glennon, R. Murciano-Goroff, E. G. Berkes, B. A. Weinberg, and J. I. Lane. 2023. "Author Correction: Women are credited less in science than men." Nature 621 (7979):E41. doi: 10.1038/s41586-023-06571-x.

25. Sá C, Cowley S, Shahrin B, Stevenson C, Su A. Disciplinary gender balance, research productivity, and recognition of men and women in academia. PLoS One. 2023;18(12):e0293080. Epub 20231214. doi: 10.1371/journal.pone.0293080. PubMed PMID: 38096215; PubMed Central PMCID: PMCPMC10720991.

26. Gaiaschi, C. and Musumeci, R. (2020). Just a Matter of Time? Women's Career Advancement in Neo-Liberal Academia. An Analysis of Recruitment Trends in Italian Universities, Social Sciences, 9(9): 163. https://doi.org/10.3390/socsci9090163

27. Gaiaschi, Camilla. Doppio Standard: Donne e carriere scientifiche nell'Italia contemporanea (p.414). 2022. Carocci editore S.p.A.. Edizione del Kindle.

28. Ferree MM, Purkayastha B (2000), Equality and Cumulative Disadvantage: Response to Baxter and Wright, in "Gender and Society", 14, 6, pp. 809-13.

29. Kim JH, Oh SS, Bae SW, Park EC, Jang SI. Gender Discrimination in the Workplace: Effects on Pregnancy Planning and Childbirth among South Korean Women. Int J Environ Res Public Health. 2019 Jul 26;16(15):2672. doi: 10.3390/ijerph16152672. PMID: 31357386; PMCID: PMC6695730.

30. Picardi, I. (2020) Labirinti di cristallo, Strutture di genere nell'accademia e nella ricerca, Milano, Franco Angeli.
