# Supplementary material for: Gender differences in the Italian academic landscape: Examining inequalities within the medical area in the last decade
Source: PLoS One. 2025 Jul 23;20(7):e0325705. doi: 10.1371/journal.pone.0325705 (PMC12286393; doi:10.1371/journal.pone.0325705)
Supplement: Attachment — Submitted filename: Point by Point Letter 2.docx [file pone.0325705.s005.docx]

Dear Editor,

We would like to thank you and the reviewers for the time and effort dedicated to evaluating our manuscript titled *"* *Gender Differences in the Italian Academic Landscape: Examining Inequalities within the Medical Area in the Last Decade"*. We greatly appreciate the insightful and constructive comments, which have helped us improve the clarity and scientific value of our work.

Below, we provide a point-by-point response to each reviewer’s comments. All changes have been incorporated in the revised version of the manuscript (in blue), which we resubmit for your consideration.

**Reviewer #1**

**Comment (C):** The manuscript has been improved substantially with full description of key topics and interpretations. I have only found a few areas where minor corrections may be required to make the article easy for the reader.

**Answer (A):** We are grateful to Reviewer 1 for this comment and for the suggested improvements. We have revised the manuscript accordingly.

**Reviewer #3**

**C:** Dear Authors, as much as I can understand Your position, I strongly advise You to use the gender definition adopted by WHO. even though You cannot adapt to the italian system You should underline this. please use all the provided references also to improve your discussion.

**A:** We thank Reviewer 3 for this comment. As recommended, we have included this point and the suggested references in the discussion section.

Once again, we sincerely thank all reviewers for their valuable contributions. We hope the revised version meets the journal’s standards and look forward to your feedback.

Kind regards,
Antonella Agodi
